# microRNA-15b modulates stress-induced premature senescence in chronic doxorubicin cardiomyopathy

**DOI:** 10.3389/fphar.2026.1924670

**Published:** 2026-10-02

**Authors:** Georthan Mancilla, Clara Quiroga, Pablo Castro, Ivonne Olmedo, Hugo Verdejo, Zully Pedrozo

**Affiliations:** 1 NIFBF, Faculty of Medicine, Institute of Biomedical Sciences, Universidad de Chile, Santiago, Chile; 2 Cardiovascular Signaling Laboratory, Division of Cardiovascular Diseases, Faculty of Medicine, Pontificia Universidad Católica de Chile, Santiago de Chile, Chile; 3 Advanced Center for Chronic Diseases (ACCDiS), Pontificia Universidad Católica de Chile and Universidad de Chile, Santiago de Chile, Chile; 4 Cardiovascular Research Unit, Cardiovascular Department, Hospital Clínico de la Universidad de Chile, Santiago de Chile, Chile

**Keywords:** cardiomyocyte, cardiotoxicity, cellular senescence, doxorubicin, miR-15b-5p, SASP

## Abstract

**Introduction:**

Doxorubicin (DOX) is the most widely used anthracycline in hematologic and solid tumors, whose clinical utility is constrained by dose-dependent cumulative cardiotoxicity that can progress to chronic heart failure. Although previously attributed to overt cardiomyocyte loss, emerging evidence indicates that stress-induced premature senescence (SIPS) constitutes the dominant mechanism of chronic cardiotoxicity at clinically relevant concentrations. SIPS is defined by permanent cell cycle arrest, activation of the p53/p21 and p16/Rb tumor suppressor axes, mitochondrial dysfunction, and a senescence-associated secretory phenotype (SASP) that sustains a pro-inflammatory myocardial milieu. Nevertheless, the post-transcriptional mechanisms that govern this process remain incompletely understood.

**Methods:**

The post-transcriptional regulation of DOX-induced SIPS was evaluated *in vitro* using neonatal rat ventricular myocytes (NRVMs) exposed to sublethal DOX (0.1 μM) with or without prophylactic miR-15b restoration via a synthetic mimetic to assess the p53/p21, p16, SASP, lysosomal, and mitochondrial responses. *In vivo*, a chronic cardiotoxicity model was established in female C57BL/6 mice receiving a cumulative dose of 30 mg/kg DOX to analyze myocardial miR-15b suppression, SIPS markers, and SASP profiles across survival cohorts.

**Results:**

Sublethal DOX treatment (0.1 μM) suppressed miR-15b expression by approximately 50% in NRVMs, resulting in post-transcriptional derepression of its mitochondrial target, SIRT4. This miR-15b loss triggered a multiparametric SIPS phenotype encompassing accumulation of γH2AX foci, p53/p21 and p16 activation, SASP induction (IL-6, IL-1β, Mcp-1 and Mmp13), lysosomal dysfunction (increased SA-β-Gal staining) and mitochondrial dysfunction. Prophylactic restoration of miR-15b via a synthetic mimetic selectively attenuated the p53/p21 axis, genomic damage foci, lysosomal dysfunction, mitochondrial dysfunction and SASP (IL-6 and IL-1β), without rescuing the p16/Rb pathway. In the female C57BL/6 chronic cardiotoxicity model, the cohort spontaneously stratified into animals completing the protocol (ND; 67%) and a premature death subgroup (PD; 33%). The PD subgroup exhibited the most profound myocardial miR-15b suppression and greater accumulation of SIPS markers, including γH2AX, p16, and reduced Lamin B. Notably, the SASP displayed distinct qualitative profiles between PD and ND animals, suggesting that SASP composition, rather than magnitude alone, determines the cardiotoxic outcome.

**Conclusion:**

Collectively, these findings position miR-15b as a key post-transcriptional regulator of DOX-induced SIPS in the cardiomyocyte, whose sustained loss associates with cardiotoxicity severity *in vivo*.

## 1 Introduction

Cancer and cardiovascular disease are the leading causes of mortality worldwide (Martínez-Sanguinetti et al., 2021; Dattani et al., 2023). As oncological survival improves, managing cardiovascular complications from antineoplastic therapies has become a major challenge in cardio-oncology (Cardinale et al., 2015; Cardinale et al., 2020). In this context, doxorubicin (DOX)—a first-line anthracycline for prevalent cancers like breast and lung (Sung et al., 2021; Douedi and Carson, 2026)—is one of the most cardiotoxic chemotherapeutic agents in clinical use. Its cumulative, dose-dependent cardiotoxicity causes a cardiomyopathy with reduced ejection fraction that can progress to overt heart failure up to 5 years post-treatment (Lefrak et al., 1973; Lipshultz et al., 1995; Lipshultz et al., 2005; Christiansen and Autschbach, 2006; McGowan et al., 2017; Cardinale et al., 2015; Cardinale et al., 2020). Consequently, guidelines define a threshold of 300 mg/m^2^ where myocardial damage increases dose-dependently up to a lifetime maximum of 550 mg/m^2^ (Douedi and Carson, 2026).

Historically, DOX cardiotoxicity was attributed to widespread cardiomyocyte apoptosis driven by oxidative stress, mitochondrial dysfunction, and topoisomerase II inhibition (Mukhopadhyay et al., 2009; Zhang et al., 2012; Pecoraro et al., 2016; Hanf et al., 2019). However, accumulating evidence demonstrates that at clinically achievable plasma concentrations (∼1 µM peak; Eksborg et al., 1985; Harahap et al., 2020), DOX predominantly triggers stress-induced premature senescence (SIPS) rather than cell death (Spallarossa et al., 2009; Meng and Lin, 2020). SIPS is characterized by irreversible cell cycle arrest via p53/p21 and p16/Rb pathways, mitochondrial dysfunction, and the secretion of a pro-inflammatory senescence-associated secretory phenotype (SASP) (Kuilman et al., 2010; Gorgoulis et al., 2019). Unlike the immunologically silent elimination of apoptotic cells, senescent cardiomyocytes remain metabolically active, exerting paracrine inflammatory effects that perpetuate pathological remodeling (Shimizu and Minamino, 2019; Chen et al., 2022). The primacy of SIPS in chronic DOX toxicity is supported by *in vivo* transcriptomic analyses showing enrichment for senescence and inflammatory signaling, but not apoptosis (Zhang et al., 2012; Zeiss et al., 2019).

Post-transcriptional regulation by microRNAs (miRNAs) is pivotal in DOX-induced SIPS (Cufí et al., 2012; Kansakar et al., 2022). By repressing target mRNAs, miRNAs act as master regulators of cell fate and senescence (Filipowicz et al., 2008; Carthew and Sontheimer, 2009; Finnerty et al., 2010; Pourrajab et al., 2015; McDonald et al., 2024). The evolutionarily conserved miR-15b family (Yue and Tigyi, 2010) modulates cardiac hypertrophy, TGF-β signaling, and post-infarction remodeling (Van Rooij et al., 2006; Hullinger et al., 2012; Tijsen et al., 2014). Validated miR-15b targets include cell cycle drivers (CCND1, CCNE1; Xia et al., 2009; Li et al., 2022), survival factors (Bcl-2, AKT; Li et al., 2020; Zhu et al., 2017), and SIRT4—a mitochondrial deacylase that suppresses oxidative phosphorylation and promotes stress-induced ROS generation (Lang et al., 2016; Martino et al., 2023). In neonatal cardiomyocytes, miR-15b restricts proliferation (Porrello et al., 2011) and functions as a “mito-miR” (Nishi et al., 2010; Dasgupta et al., 2015) that directly controls cardiac bioenergetics (Pantaleão et al., 2024; Zhang et al., 2024).

The link between miR-15b and senescence was established in human dermal fibroblasts, where miR-15b loss derepresses SIRT4, driving mitochondrial ROS generation, loss of membrane potential (ΔΨm), and SASP activation (Lang et al., 2016). In cardiomyocytes, miR-15b restoration attenuates p53 and pro-fibrotic signaling in diabetic cardiomyopathy (Rawal et al., 2017), while extracellular vesicles enriched in miR-15b rejuvenate senescent cells (Yu et al., 2023). However, the role of the miR-15b/SIRT4 axis in DOX-induced cardiomyocyte SIPS and its impact on chronic cardiotoxicity severity *in vivo* remain unknown.

A critical ambiguity in the literature is the discordant response of miR-15b to DOX: high concentrations (6 µM) upregulate miR-15b during acute toxicity (Wan et al., 2019; Wan et al., 2021), whereas lower, senescence-inducing doses trigger sustained downregulation (Holmgren et al., 2016). This discordance likely reflects a dose-dependent divergence in cardiomyocyte fate: at supra-clinical, an apoptotic dose, miR-15b upregulation represses BCL-2 to potentiate cell death (Li et al., 2020); at sublethal, clinically relevant doses, sustained miR-15b loss derepresses SIRT4 and CCND1, permitting SIPS (Lang et al., 2016; Li et al., 2022). This dual-response framework underpins the rationale of our study.

Here, we propose that DOX-mediated miR-15b suppression at clinically relevant concentrations acts as a permissive mechanism for cardiomyocyte SIPS, contributing to chronic cardiac dysfunction, and that myocardial miR-15b levels inversely predict cardiotoxicity severity *in vivo*. To test this hypothesis, we combined *in vitro* gain-of-function experiments in neonatal rat ventricular myocytes (NRVMs) with a female murine chronic cardiotoxicity model that recapitulates patient phenotypic heterogeneity (Cardinale et al., 2020). Our findings identify the miR-15b/SIRT4 axis as a conserved regulator of cardiomyocyte SIPS, highlighting its potential translational relevance in anthracycline cardiomyopathy.

## 2 Materials and methods

### 2.1 Adult animal and chronic cardiotoxicity model

Female C57BL/6 mice (10 weeks old) were obtained from the Central Animal Facility, Faculty of Medicine, Universidad de Chile (Santiago, Chile). Animals were maintained under standard 12:12 h light/dark conditions at 22 °C ± 1 °C, with *ad libitum* access to food and water. To induce chronic cardiotoxicity, mice received intraperitoneal injections of doxorubicin (DOX; NDC 0069-3030-20; Pfizer, New York, NY, United States) at 6 mg/kg once weekly for 5 consecutive weeks (cumulative dose: 30 mg/kg), followed by a 5-week monitoring period. Control of animals received intraperitoneal saline. All procedures complied with the World Medical Association Statement on Animal Use in Biomedical Research and were approved by the Institutional Committee for the Care and Use of Animals of the Universidad de Chile (Approval No. CBA 1173 FMUCH). Euthanasia was performed by inhalation of isoflurane.

### 2.2 Cardiomyocyte culture experiments

Neonatal rat ventricular myocytes (NRVMs) were isolated from 1–3-day-old Sprague-Dawley rat hearts (Maass and Buvoli, 2007). Following enzymatic digestion, cardiomyocytes were cultured on 2% gelatin-precoated plates (Winkler Ltda., Santiago, Chile) at 37 °C in 5% CO_2_ in maintenance medium: Minimum Essential Medium Eagle (M4655; MEM; Sigma-Aldrich, St. Louis, MO, United States) supplemented with 5% fetal bovine serum (FBS; 10099141; Gibco, Grand Island, NY, United States), 1% ampicillin/streptomycin, and 100 µM 5-bromo-2-deoxyuridine (B5002; Sigma-Aldrich, St. Louis, MO, United States). Stimulations were performed 24 h after plating. To induce senescence, NRVMs were treated with 0.1 µM DOX (NDC 0069-3030-20; Pfizer, New York, NY, United States) or vehicle (saline) for 24 h (Spallarossa et al., 2009). All procedures were approved by the Institutional Committee for the Care and Use of Animals of the Universidad de Chile (Approval No. CBA 1388 FMUCH).

### 2.3 Cell transfection

A total of 7.5 × 10^5^ cells were seeded in 35-mm plates to 70% confluence. Cells were transfected with mirVana miRNA Mimic Negative Control #1 (4464058; Thermo Fisher Scientific, Waltham, MA, United States) or mirVana Mimic - miR-15b mimetic (Assay ID MC10904, #4464066; Thermo Fisher Scientific, Waltham, MA, United States) at final concentration of 6.25, 12.5, 25 or 50 nM using FuGENE (E2311; Promega, Madison, WI, United States) according to the manufacturer’s instructions. Transfections were performed 24 h before DOX stimulation.

### 2.4 RNA/miRNA isolation and quantification

Total RNA was extracted from NRVM cultures or cardiac ventricular tissue using the Trizol-chloroform method (Thermo Fisher Scientific; Chomczynski and Mackey, 1995). RNA concentration and purity was measured with a NanoDrop-1000 spectrophotometer (Thermo Fisher Scientific, Waltham, MA, United States). For mRNA analysis, cDNA was synthesized from 1 µg total RNA using M-MLV reverse transcriptase (M1705; Promega, Madison, WI, United States) with random primer. Total miRNA was quantified using the Qubit microRNA Assay Kit (Thermo Fisher Scientific, Waltham, MA, United States), and miRNA cDNA was prepared with the miScript II RT Kit (218161; Qiagen, Hilden, Germany). RT-qPCR was performed on a StepOne Real-Time PCR System (Thermo Fisher Scientific, Waltham, MA, United States) using SensiFAST Hi-ROX probes (BIO-82020; Meridian Bioscience, Cincinnati, OH, United States). miRNA primers were designed with miRprimer2 software (Busk, 2014). Relative expression was calculated by the ΔΔCt method (Livak and Schmittgen, 2001), using Pabpn or 18S rRNA as endogenous controls for mRNA and RnU6b for miRNA. Primer sequences are listed in Table 1.

**TABLE 1:** Primers used for RT-qPCR mRNA primers were designed with Primer3 (NCBI) and validated by BLAST against *Rattus norvegicus* and *Mus musculus* genomes. miRNA primers were designed with miRprimer2 software (Busk, 2014) using miRBase mature sequences (Accession: MIMAT0000124/MIMAT0000784 for rno/mmu-miR-15b-5p). Amplification efficiency validated by serial dilution (E = 90–110%). *miRNA primers designed with miRprimer2 on miRBase sequences.

Gene/RNA	Species	Forward (5'→3′)	Reverse (5'→3′)
*p53 (exons 7–8)*	Rat	GTG​GTA​CCG​TAT​GAG​CCA​CC	AGG​CAC​AAA​CAC​GAA​CCT​CA
*p21*	Rat	GAT​CCT​GGT​GAT​GTC​CGA​CC	GCG​AAG​TCA​AAG​TTC​CAC​CG
*p16*	Rat	TCG​TGC​GGT​ATT​TGC​GGT​AT	TAG​TCT​CGC​GTT​GCC​AGA​AG
*Il-6*	Rat	CCA​CGA​GGA​TCA​GTA​CGG​AAG	TCT​CAG​CCT​GTG​ACT​TCC​ATT​T
*Sirt4*	Rat	CGG​AAG​TGC​TGA​GGT​CAT​CA	CCT​GTG​ACG​TTG​GAG​AAG​CC
*Ccnd1*	Rat	AAC​TTC​CTC​TCC​TGC​TAC​CG	GAG​ATG​GAG​GGA​GTC​CTT​GT
*Acox1*	Rat	GCA​TCT​CGG​ACC​CTG​AAG​AA	GGC​GTA​GGT​GCC​AAT​TAT​CTG
*Gata4*	Rat	CTG​CGA​GAC​ACC​CCA​ATC​TC	CCA​CAG​GCG​TTG​CAT​AGG​TA
*Smc4*	Rat	AGC​CAT​GGG​ATT​GAC​TTA​GAC​C	TGT​TCA​GTC​TGG​CCT​TTT​GGT
*Bax*	Rat	CGCACGTCTGCGGGGA	ATG​ATC​TGT​TCA​GAG​CTG​GTG​G
*Il-1β*	Rat	TGT​CTG​AAG​CAG​CTA​TGG​CAA	GGG​CTT​GGA​AGC​AAT​CCT​TAA​TC
*Tgf- β*	Rat	ACT​GAT​ACG​CCT​GAG​TGG​CT	AGC​CCT​GTA​TTC​CGT​CTC​CTT
*Mcp-1*	Rat	CAG​CCA​GAT​GCA​GTT​AAT​GCC	AGC​CGA​CTC​ATT​GGG​ATC​AT
*Mmp13*	Rat	TGC​TGC​ATA​CGA​GCA​TCC​AT	CCC​CGT​GTC​CTC​AAA​GTG​AA
*Bcl-2*	Rat/mouse	ATG​CCT​TTG​TGG​AAC​TAT​ATG​GC	GGT​ATG​CAC​CCA​GAG​TGA​TGC
*p53*	Mouse	AGA​GAC​CGC​CGT​ACA​GAA​GA	CTG​TAG​CAT​GGG​CAT​CCT​TT
*p21*	Mouse	CCT​GGT​GAT​GTC​CGA​CCT​G	CCA​TGA​GCG​CAT​CGC​AAT​CA
*p16*	Mouse	CCCAACGCCCCGAACT	GCA​GAA​GAG​CTG​CTA​CGT​GAA
*Mcp-1*	Mouse	GCA​TCC​ACG​TGT​TGG​CTC​A	CTC​CAG​CCT​ACT​CAT​TGG​GAT​CA
*Il-1β*	Mouse	TCC​TCG​GCC​AAG​ACA​GGT​CGC​T	CCC​CCA​CAC​GTT​GAC​AGC​TAG​GT
*Il-6*	Mouse	CCA​GAG​ATA​CAA​AGA​AAT​GAT​GG	G ACT​CCA​GAA​GAC​CAG​AGG​AAA​T
*Tgf-β*	Mouse	GCT​GCT​GAC​CCC​CAC​TGA​T	GAG​CGC​TGA​ATC​GAA​AGC​C
*Ccl8*	Mouse	TAA​GGC​TCC​AGT​CAC​CTG​CT	TCT​GGA​AAA​CCA​CAG​CTT​CC
*Bnp*	Mouse	GAG​GTC​ACT​CCT​ATC​CTC​TGG	GCC​ATT​TCC​TCC​GAC​TTT​TCT​C
*Sur2a*	Mouse	CCT​TCG​GCT​CTC​GAC​TTC​TA	ACT​CGA​CCC​AAG​CAA​ATT​GT
*Atrogin1*	Mouse	CAG​TTT​GAC​CGT​GAG​GAG​GG	CAG​ACT​TCG​GCA​GCC​TTC​AG
*Murf1*	Mouse	TGT​CTG​GAG​GTC​GTT​TCC​GT	ATC​CAG​CAG​CTT​GCT​GAA​CA
*Acox1*	Mouse	GGA​TCA​GCC​AGA​AAG​GAG​GG	CAT​CAT​AGC​GGC​CGA​GAA​CA
*Sirt4*	Mouse	ATT​CCC​GCT​GTG​GAG​AGT​TG	TTC​AGA​GTT​GGA​GCG​GCA​TT
*Ccnd1*	Mouse	TCT​CCT​GCT​ACC​GCA​CAA​C	ATG​GAG​GGG​GTC​CTT​GTT​TAG
*Pabpn*	Rat/mouse	GTT​GGC​AAT​GTG​GAC​TAT​GG	TAT​GCA​AAC​CCT​TTG​GGA​TG
*18S rRNA*	Mouse	CGGACAGGATTGACAGAT	CAA​ATC​GCT​CCA​CCA​ACT​A
*Smc4*	Mouse	AGA​GAC​AGA​CGC​TGG​AGA​GTA​GTG	TTG​CTT​CCA​CCG​CCA​CTC​ATT​G
*miR-15b-5p*	Rat/mouse	AGT​AGC​AGC​ACA​TCA​TGG​T	TCC​AGT​TTT​TTT​TTT​TTT​TTG​TAA​ACC
*pre-miR-15b*	Rat/mouse	AGC​AGC​ACA​TCA​TGG​TTT​ACA	TAA​TGA​TTC​GCA​TCT​TGA​CTG​TAG​C
*rnU6b*	Rat	CCT​GCT​TCG​GCA​GCA​CAT​A	AAC​GCT​TCA​CGA​ATT​TGC​GT

The asterisk (*) indicates miR-15b-5p.

### 2.5 Protein extraction and analysis

Protein changes were assessed by Western blot. Cardiac tissue samples were homogenized in T-PER lysis buffer (78510; Thermo Fisher Scientific, Waltham, MA, United States) containing protease and phosphatase inhibitor cocktails (539134 and 524627; Merck Millipore, Burlington, MA, United States). Proteins were resolved by SDS-PAGE and transferred to PVDF membranes (IPVH00010; Merck Millipore, Burlington, MA, United States). Membranes were blocked and incubated with primary antibodies: anti-γH2AX (1:1,000; MA1-2022, Thermo Fisher Scientific, Waltham, MA, United States), anti-p53 (1:1,000; 2,524; Cell Signaling Technology, Danvers, MA, United States), anti-p21 (1:1,000; ab109199; Abcam, Cambridge, United Kingdom), anti-Lamin B (1:2,500; ab16048; Abcam, Cambridge, United Kingdom), and anti-GAPDH (1:40,000; G9545; Merck Millipore, Burlington, MA, United States), followed by HRP-conjugated secondary antibodies (1:5,000; 7,074 and 7,076; Cell Signaling Technology, Danvers, MA, United States). Chemiluminescence was detected with ECL solution, digitized using a C-DiGit Blot Scanner (LI-COR Biosciences, Lincoln, NE, United States), and quantified with Image Studio Lite v5.2. Protein signals were normalized to GAPDH. When membranes were stripped and reprobed, complete erasure of prior chemiluminescent signal was confirmed by re-incubating with secondary antibody alone and imaging prior to primary antibody re-probing.

### 2.6 Immunofluorescence

Cells on gelatin-coated glass coverslips were fixed with 4% paraformaldehyde for 15 min at room temperature, permeabilized with 0.1% Triton X-100 for 10 min, and blocked with 3% bovine serum albumin (BSA) in PBS for 1 h. Samples were incubated overnight at 4 °C with primary antibodies: anti-γH2AX (1:1,000; MA1-2022, Thermo Fisher Scientific, Waltham, MA, United States), anti-p53 (1:1,000; ab131442; Abcam, Cambridge, United Kingdom) and anti-p21 (1:1,000; ab109199; Abcam, Cambridge, United Kingdom), followed by fluorochrome-conjugated secondary antibodies (A-11034 and A-11004; Thermo Fisher Scientific) for 1 h at room temperature. Nuclei were counterstained with Hoechst 33342 (1:1,000; H3570; Thermo Fisher Scientific, Waltham, MA, United States). Coverslips were mounted with Fluoromount-G (0100-001; SouthernBiotech, Birmingham, AL, United States) and imaged on a Nikon C2+ spectral confocal microscope (Nikon Instrument, Tokyo, Japan) using ×63 and ×40 oil-immersion objectives. Image analysis was performed with CellProfiler v4.0. Image acquisition and downstream quantitative analyses were performed without blinding to experimental group assignments.

### 2.7 SA-β-gal staining

NRVMs were washed twice with PBS, fixed in 2% formaldehyde/0.2% glutaraldehyde in PBS for 5 min at room temperature, and washed twice again with PBS. Cells were then incubated at 37 °C (in a non-CO_2_ incubator) for 72 h with freshly prepared SA-β-Gal staining solution containing 1 mg/mL X-gal, 40 mM citric acid/sodium phosphate buffer (pH 6.0), 5 mM potassium ferrocyanide, 5 mM potassium ferricyanide, 150 mM NaCl, and 2 mM MgCl_2_. Development of blue perinuclear precipitate was monitored under bright-field microscopy. Images were acquired using ×10 magnification, and the percentage of SA-β-Gal-positive cells was quantified by counting positively stained cells relative to the total number of cells per field, in 5 random fields per condition.

### 2.8 Cytotoxicity assay

Cytotoxicity was assessed by measuring lactate dehydrogenase (LDH) release into culture medium using the CytoTox 96® Non-Radioactive Cytotoxicity Assay kit (G1780; Promega, Madison, WI, United States), according to the manufacturer’s instructions.

### 2.9 Flow cytometry

Apoptosis was assessed using the Annexin V-FITC/propidium iodide apoptosis detection kit (ab14085; Abcam, Cambridge, United Kingdom) according to the manufacturer’s instructions. For mitochondrial membrane potential (ΔΨm), NRVMs were incubated with 0.2 µM TMRE (21426; Cayman Chemical, Ann Arbor, MI, United States) for 30 min at room temperature. For mitochondrial reactive oxygen species measurement, cells were loaded with 1 µM MitoSOX (M36008; Thermo Fisher Scientific, Waltham, MA, United States) for 30 min at room temperature. In all experiments doublets were excluded by sequential gating on FSC-A vs. FSC-H, followed by gating on the main cell population using FSC-A vs. SSC-A to exclude debris. A minimum of 20,000 events were acquired per sample. Data were acquired on a BD FACSCanto II flow cytometer (BD Biosciences, San Jose, CA, United States).

### 2.10 Statistical analysis

Data were analyzed and plotted using GraphPad Prism v8.0.2. Dataset normality was assessed using the Shapiro–Wilk test. All data are presented as mean ± standard deviation (SD). Differences between two groups were evaluated with Student’s t-test for normally distributed data or the Mann–Whitney U test for non-parametric data. Comparisons among three or more groups were performed using one-way ANOVA followed by Tukey’s *post hoc* test. For experiments with two independent variables, two-way ANOVA was applied. Survival curves were compared by the log-rank (Mantel–Cox) test. A p-value < 0.05 was considered statistically significant.

## 3 Results

### 3.1 Sublethal DOX exposure induces SIPS in cardiomyocytes *in vitro* without triggering programmed cell death

To identify a DOX concentration that induces SIPS without engaging canonical programmed cell death pathways, primary NRVM cultures were exposed for 24 h to increasing DOX concentrations (0, 0.1, 0.5, 1 and 5 µM) and cytotoxicity was quantified by LDH release into the culture medium. Significant cytotoxic effects were observed only at concentrations ≥ 0.5 µM, whereas 0.1 µM DOX produced LDH release levels statistically indistinguishable from vehicle-treated controls (Figure 1A). Flow cytometric analysis using Annexin-V/PI staining (Figure 1B) confirmed that 0.1 µM DOX did not increase early apoptosis (Annexin-V+/PI−; Figure 1C). Although a modest increase in the late apoptotic/secondary necrotic fraction was detected (Figure 1D), this did not constitute significant cardiomyocyte loss. At a transcriptional level, the *Bax/Bcl-2* mRNA ratio, a canonical readout of pro- vs. anti-apoptotic signaling balance, remained unchanged between groups (Figure 1E), consistent with the absence of an active pro-apoptotic transcriptional program. Collectively, this evidence establishes that 0.1 µM DOX for 24 h does not engage programmed cell death in NRVMs and was selected as the reference concentration for all subsequent *in vitro* experiments.

**FIGURE 1:**
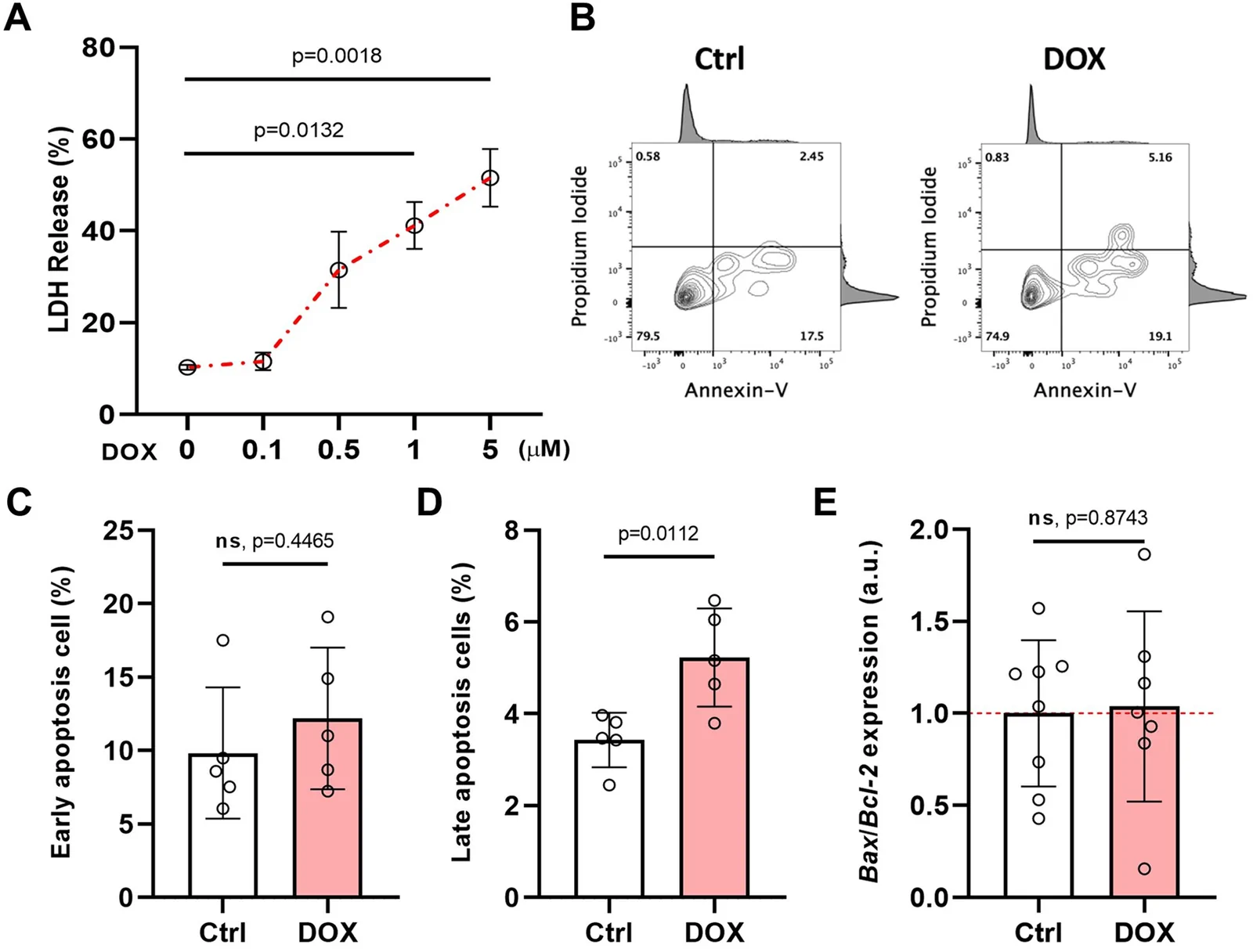
Cytotoxicity profiling and apoptosis assessment in DOX-treated neonatal rat ventricular myocytes (NRVMs). **(A)** Dose–response curve of lactate dehydrogenase (LDH) release in NRVMs exposed to DOX (0–5 µM) for 24 h; one-way ANOVA with Tukey’s *post hoc* test (n = 3). **(B)** Representative flow cytometry contour plots of Annexin-V-FITC/propidium iodide (PI) staining in vehicle-treated (Ctrl) and 0.1 µM DOX-treated NRVMs for 24 h. **(C)** Quantification of early apoptosis (Annexin-V+/PI−) by flow cytometry (n = 5). **(D)** Quantification of late apoptosis/secondary necrosis (Annexin-V+/PI+) by flow cytometry (n = 5). **(E)** *Bax*/*Bcl-2* mRNA ratio by RT-qPCR (n = 7–8). Data are presented as mean ± standard deviation (SD). p < 0.05 vs. the reference control (Ctrl, or DOX 0 µM) was considered statistically significant difference. ns: statistically non-significant difference.

Using the sublethal concentration established above, we next assessed whether DOX redirected the cardiomyocyte toward a senescent phenotype. DOX-exposed NRVMs exhibited activation of the γH2AX/p53/p21/p16 axis during the SIPS response (Figure 2). Confocal immunofluorescence analysis revealed a marked increase in the nuclear γH2AX foci, a marker of DNA double-strand breaks, with the percentage of γH2AX-positive cells increasing from approximately 20% in controls to more than 70% following DOX treatment (Figures 2A,B). This DNA damage response was accompanied by coordinated activation of the p53/p21 tumor suppressor axis. Using exon-specific RT-qPCR analysis, our results revealed an increase in transcripts covering the exon 7–8 region (Figure 2C), whereas immunofluorescence showed a significant increase in mean cellular p53 fluorescence intensity (Figures 2D,E). This pattern is consistent with differential representation of p53 transcript variants under genotoxic stress. Consistent with p53 activation, its canonical transcriptional target p21 (CDKN1A) was significantly induced at both mRNA and protein levels (Figures 2F–H). *p16* (CDKN2A) mRNA was also significantly upregulated in DOX-treated cells (Figure 2I), consistent with parallel activation of the p16/Rb axis. On the other hand, we observed that DOX treatment increased the mRNA levels of *Il-6*, *Il-1β, Mcp-1 and Mmp13* (Figures 3A–D), without changes in *Tgf-β* (Figure 3E). To confirm SIPS engagement at the canonical enzymatic level, SA-β-gal activity was assessed. NRVMs exposed to DOX displayed a marked increase in the proportion of SA-β-gal-positive cells relative to controls (Figures 3F,G). Mitochondrial assessment revealed elevated mitochondrial superoxide levels by MitoSOX fluorescence (Figures 3H,I) and a significant reduction in the mitochondrial membrane potential (ΔΨm) measured by TMRE fluorescence normalized to MitoTracker (Figure 3J). These data establish that 0.1 µM DOX for 24 h is sufficient to trigger a comprehensive SIPS phenotype in NRVMs, encompassing DNA damage, activation of both p53/p21 and p16/Rb axes, SASP induction, and mitochondrial dysfunction, in the absence of programmed cell death.

**FIGURE 2:**
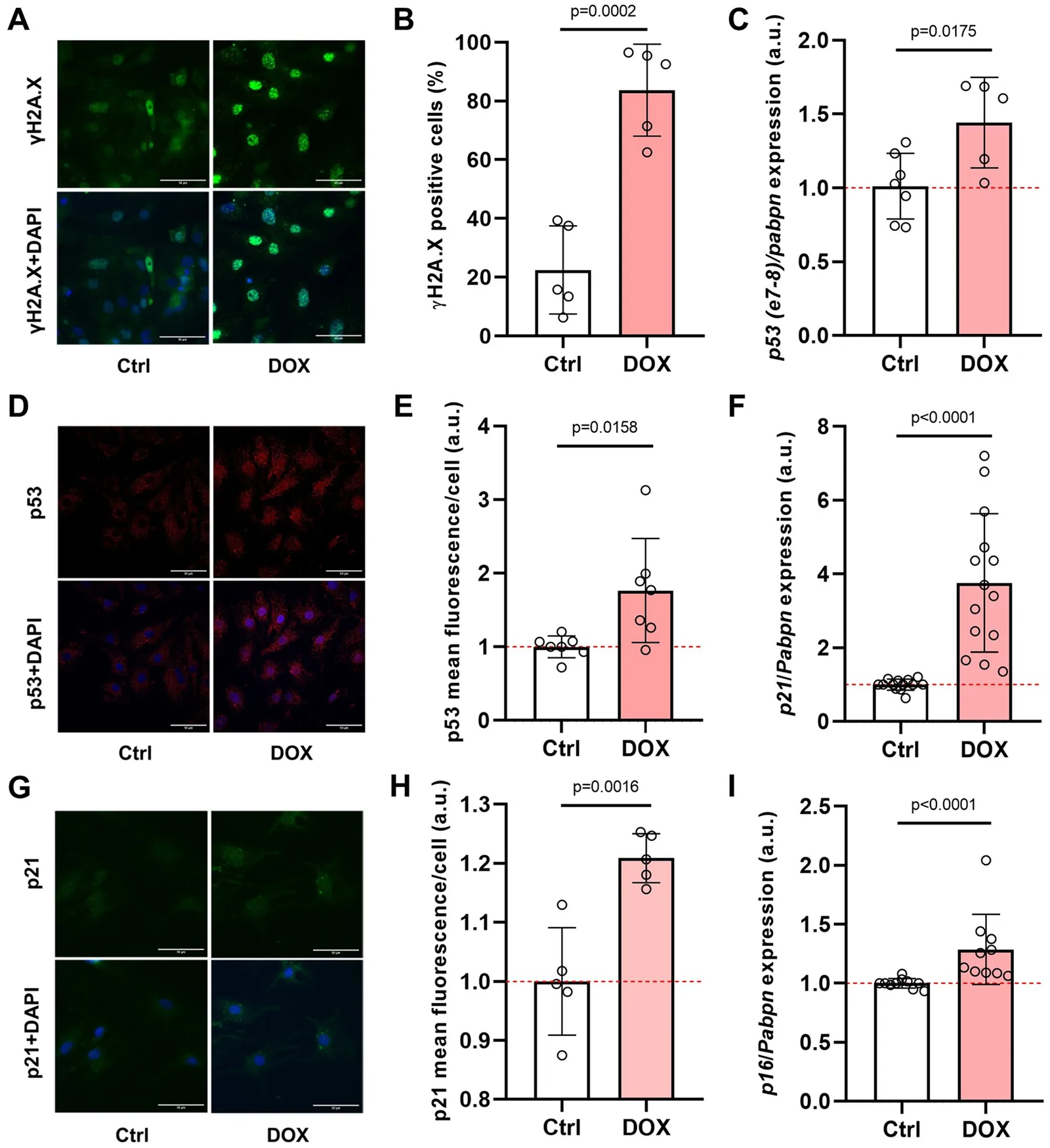
Activation of the γH2AX/p53/p21/p16 axis in DOX-treated NRVMs for 24 h. **(A)** Representative confocal immunofluorescence images of γH2AX in NRVMs treated with vehicle or 0.1 µM DOX. **(B)** Quantification of the percentage of γH2AX-positive cells (n = 5). *p53* mRNA levels spanning exons 7–8 (n = 5–7). **(C)** Quantified by RT-qPCR and normalized to *Pabpn*. **(D,E)** Representative immunofluorescence images and quantification of mean nuclear p53 fluorescence intensity (n = 7). **(F)** *p21* mRNA by RT-qPCR (n = 14). **(G,H)** Representative immunofluorescence images and quantification of mean nuclear p21 fluorescence intensity (n = 5). **(I)** *p16* mRNA by RT-qPCR. mRNA levels were normalized to *Pabpn* mRNA. Data are presented as mean ± standard deviation (SD). p < 0.05 vs. the reference control (Ctrl) was considered statistically significant difference.

**FIGURE 3:**
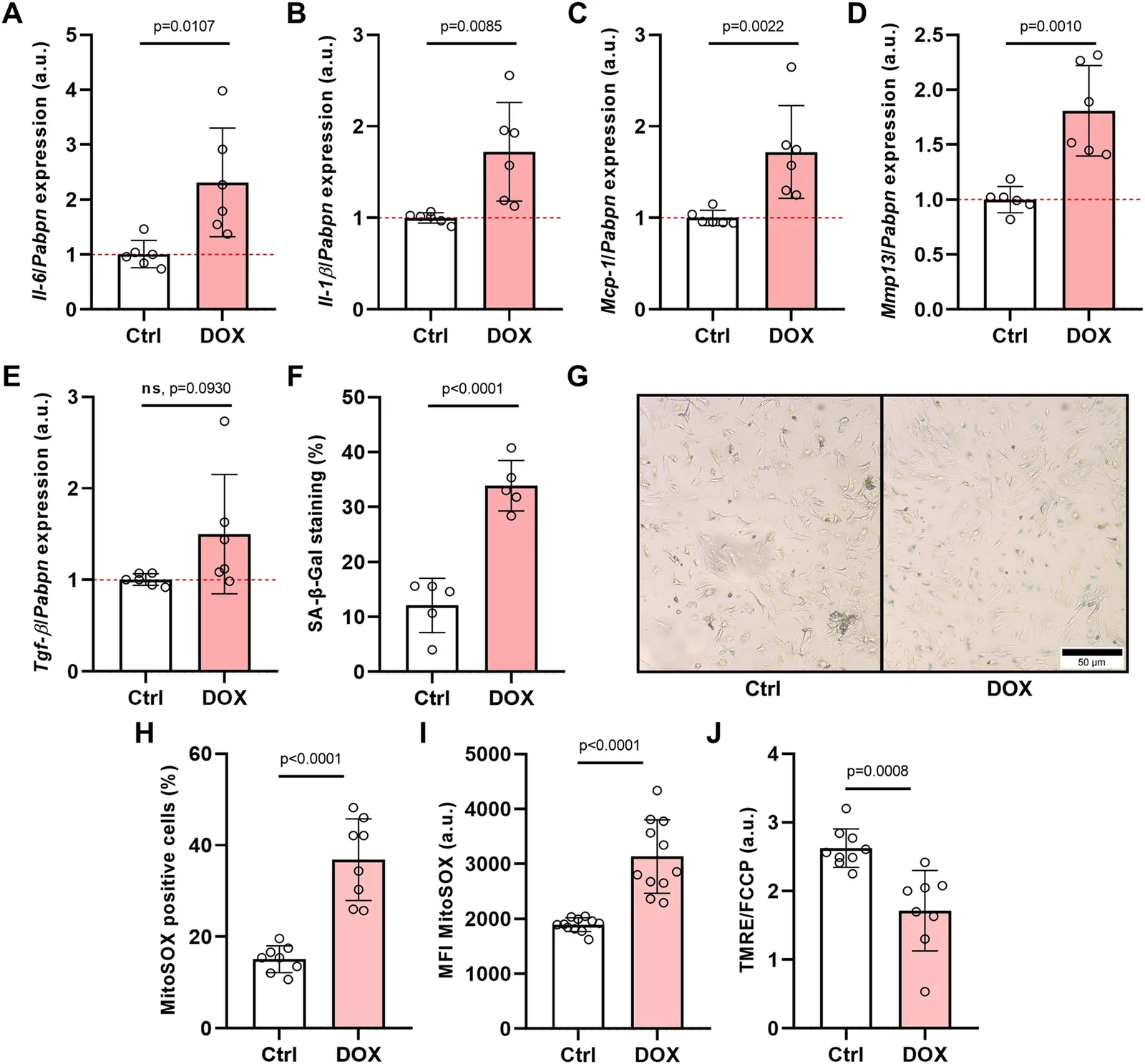
Markers of senescence in DOX-treated NRVMs for 24 h. **(A)** *Il-6* mRNA (n = 6), **(B)** *Il-1* β mRNA (n = 6), **(C)** *Mcp-1* mRNA (n = 6), **(D)** *Mmp13* mRNA (n = 6), and **(E)** *Tgf-* β mRNA (n = 6) were measured by RT-qPCR. **(F,G)** Quantification of the Lysosomal function by peri-nuclear β -Gal staining (n = 5). **(H,I)** Quantification of the MitoSOX by flow cytometry (mitochondrial superoxide) (n = 8). **(J)** TMRE reflecting mitochondrial membrane potential (ΔΨm), by flow cytometry (n = 8). mRNA levels were normalized to *Pabpn* mRNA. Data are presented as mean ± standard deviation (SD). p < 0.05 vs. the reference control (Ctrl) was considered statistically significant difference. ns: statistically non-significant difference.

### 3.2 DOX suppresses endogenous miR-15b expression and causes post-transcriptional derepression of SIRT4 in cardiomyocytes

Given the role of microRNAs in the post-transcriptional regulation of senescence, we investigated whether DOX treatment altered miR-15b homeostasis in NRVMs. RT-qPCR analysis revealed an approximately 50% reduction in mature miR-15b expression in DOX-treated cells relative to vehicle-treated controls (Figure 4A). To evaluate whether this suppression has a transcriptional component, we quantified the levels of SMC4, the host gene harboring the intron from which pre-miR-15b originates, which showed a concordant decrease (Figure 4B). Similarly, pre-miR-15b levels exhibited a decrease (Figure 4C).

**FIGURE 4:**
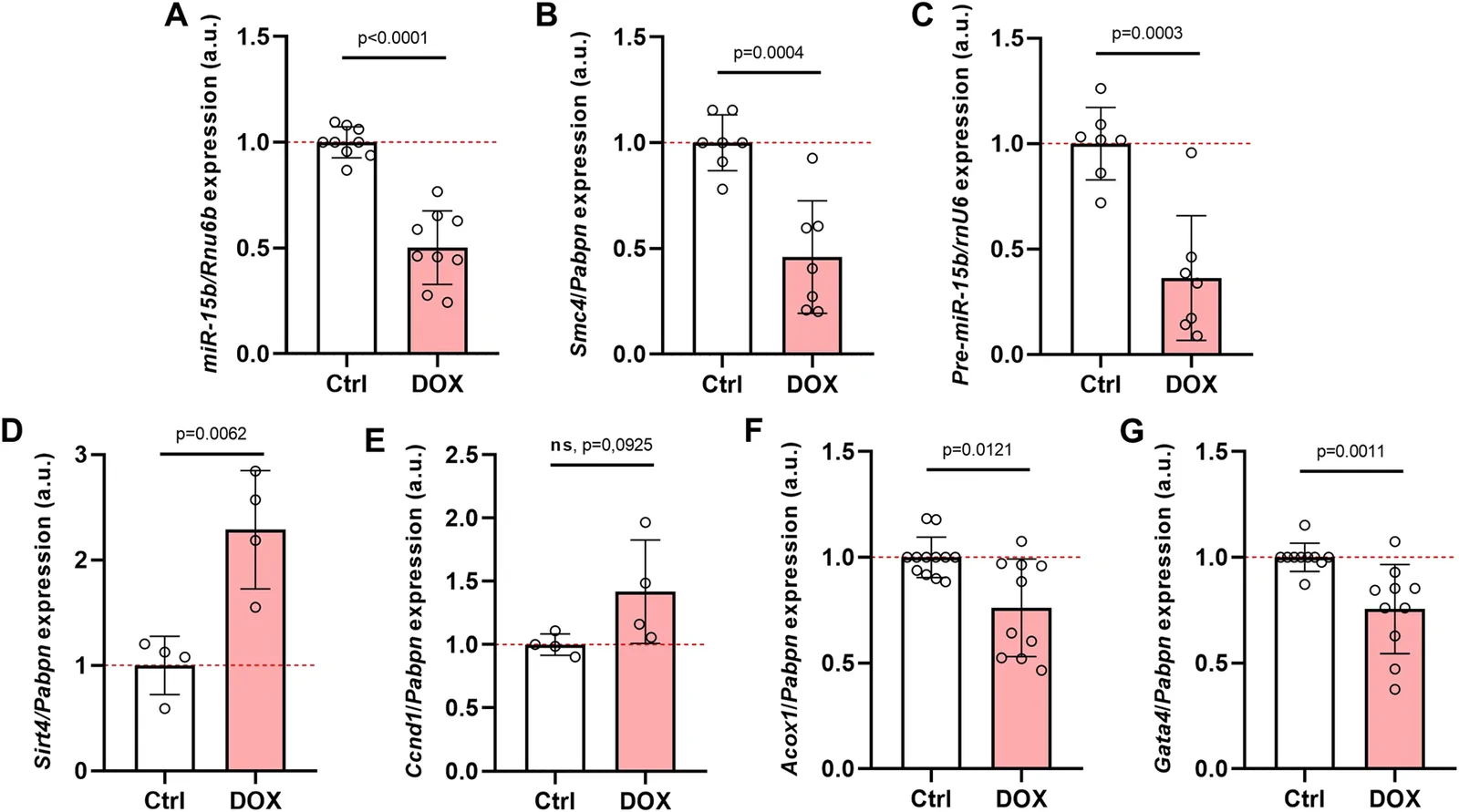
miR-15b and validated target mRNA expression in DOX-treated NRVMs. **(A)** Mature miR-15b expression normalized to *RnU6b* (n = 9), **(B)** *Smc4* mRNA (host gene containing the pre-miR-15b intron, n = 7), **(C)** Pre-miR-15b expression normalized to *RnU6b* (n = 7), **(D)** *Sirt4* mRNA (n = 4), **(E)** *Ccnd1* mRNA (n = 4), **(F)** *Acox1* mRNA (n = 10) and, **(G)** *Gata4* mRNA (n = 10), were measured by RT-qPCR. mRNA levels were normalized to *Pabpn* mRNA. Data are presented as mean ± standard deviation (SD). p < 0.05 vs. the reference control (Ctrl) was considered statistically significant difference. ns: statistically non-significant difference.

To evaluate the functional consequences of miR-15b suppression, we quantified the mRNA levels of previously validated targets: *Sirt4, Ccnd1, Acox1*, and *Gata4*. DOX treatment produced a significant increase in *Sirt4* mRNA (Figure 4D) and a non-significant trend toward *Ccnd1* elevation (p = 0.0925; Figure 4E), both consistent with post-transcriptional derepression following miR-15b loss. In contrast, *Acox1* and *Gata4* mRNAs were significantly reduced (Figures 4F,G), suggesting that, for these targets, regulatory mechanisms independent of miR-15b repression predominate under DOX treatment. These findings establish a mechanistic link between DOX-induced miR-15b suppression and SIRT4 post-transcriptional derepression, consistent with a contribution of this regulatory axis to the mitochondrial dysfunction observed in DOX-treated cardiomyocytes.

### 3.3 Prophylactic miR-15b restoration partially rescues the SIPS phenotype in cardiomyocytes treated with DOX

To investigate the causal role of miR-15b in DOX-induced SIPS, we optimized the transfection protocol of a miR-15b mimetic (M) in NRVMs. LDH cytotoxicity assays demonstrated that concentrations up to 50 nM did not significantly increase cell death relative to the transfection control (TC), while 100 nM produced statistically significant LDH release (Figure 5A). Successful intracellular delivery was confirmed by RT-qPCR, with 12.5 and 25 nM doses producing a significant increase in mature miR-15b levels above TC baseline (Figure 5B). Based on these dose-response profiles, 25 nM (hereafter M, 25 pmol per 10^6^ cells) was selected as the highest non-cytotoxic dose for gain-of-function experiments. The combined transfection and DOX (D) treatment protocol did not induce additional cytotoxicity in any of the four experimental groups: TC, TC + D, M, and M + D (Figure 5C).

**FIGURE 5:**
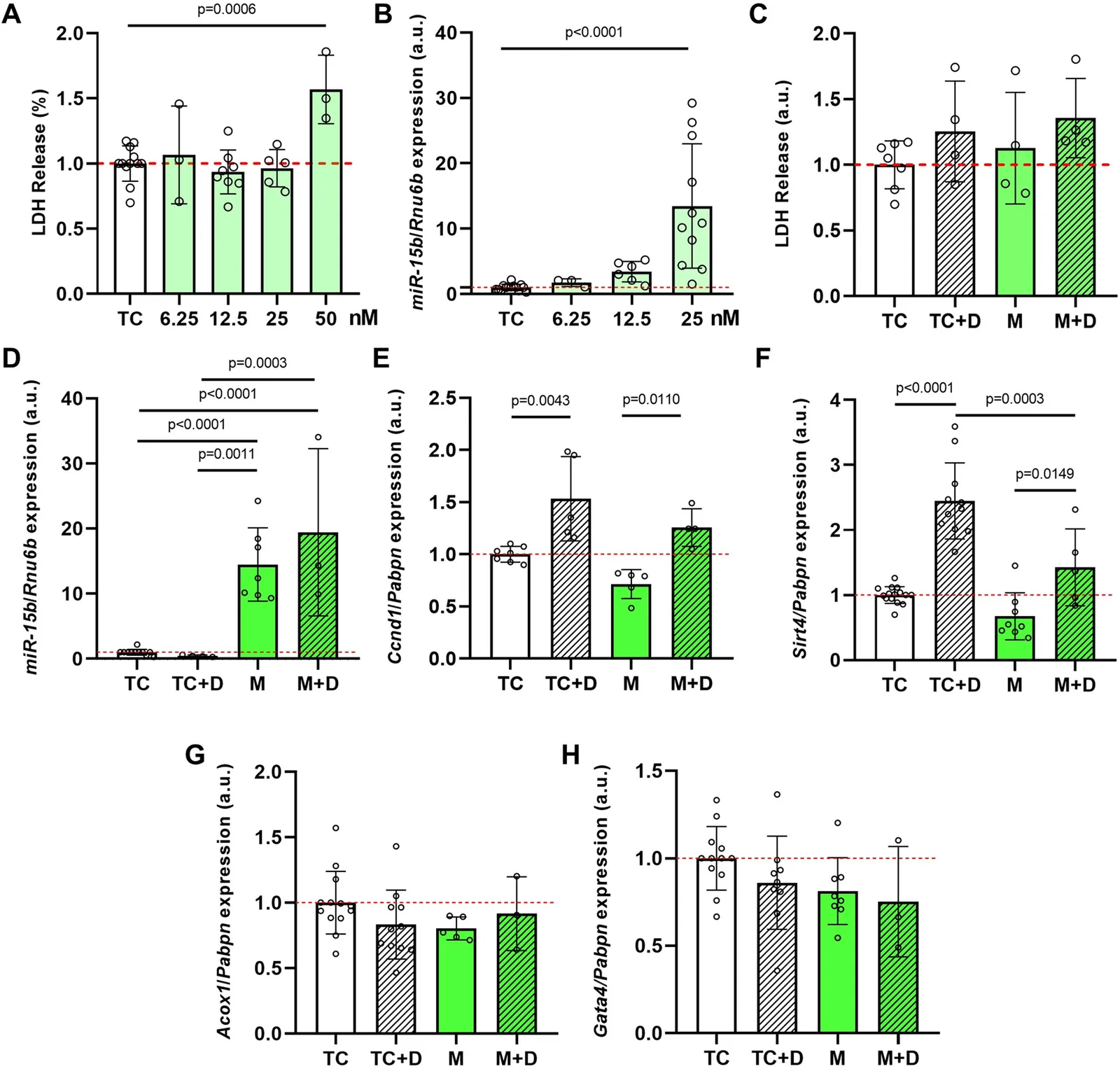
Dose optimization and functional validation of the miR-15b mimetic transfection protocol in NRVMs. **(A)** LDH cytotoxicity assay at increasing mimetic doses (6.25–50 nM) relative to TC (n = 3–12). **(B)** Mature miR-15b/*RnU6b* by RT-qPCR after transfection with 6.25, 12.5, or 25 nM mimetics, relative to TC (n = 3–11). **(C)** LDH cytotoxicity assay (n = 4–7). **(D)** Mature miR-15b/*RnU6b* by RT-qPCR (n = 3–11). **(E)** *Ccnd1* mRNA (n = 4–7), **(F)** *Sirt4* mRNA (n = 5–15), **(G)** *Acox1* mRNA (n = 3–13), and **(H)** *Gata4* mRNA (n = 3–12) were measured by RT-qPCR. mRNA levels were normalized to *Pabpn* mRNA. Data are presented as mean ± standard deviation (SD). Comparisons among 4 groups were evaluated by one-way ANOVA followed by Tukey’s *post hoc* test. p < 0.05 was considered statistically significant difference. TC: transfection control; M: miR-15b mimetic (25 nM); TC + D: DOX (0.1 µM): M + D: combined mimetic + DOX.

Functional validation confirmed that M pre-transfection elevated miR-15b levels and prevented DOX-induced miR-15b suppression in M + D cardiomyocytes (Figure 5D). Regarding validated miR-15b targets, *Ccnd1* was not significantly modified by the mimetic under basal conditions; although DOX treatment elevated its levels, this increase was not prevented by pre-transfection in M + D cells (Figure 5E). In contrast, *Sirt4* displayed a bidirectional regulatory pattern consistent with miR-15b function (Figure 5F): DOX induced significant *Sirt4* derepression in TC + D cells; and the M + D combination partially but significantly reversed DOX-induced *Sirt4* derepression. These findings confirm that the transfected mimetic is functionally active in cardiomyocytes, capable of suppressing endogenous target transcripts, most notably *Sirt4*. On the other hand, although *Acox1* (Figure 5G) and *Gata4* (Figure 5H) are validated targets of miR-15b, they did not display significant changes under this treatment regimen.

M pre-transfection significantly attenuated DOX-induced accumulation of nuclear γH2AX foci compared to TC + D cells (Figures 6A,B). At the level of the p53/p21 axis, miR-15b restoration significantly prevents the DOX-induced increase in p53 protein levels (Figures 6C,D) and *p53* mRNA expression (Figure 6E). While *p21* mRNA showed partial attenuation (Figure 6F), this did not translate to a significant change at the protein level (Figures 6G,H). In contrast, the effect of M pre-transfection was entirely absent at the level of *p16* mRNA: the DOX-induced *p16* increase was not modified by miR-15b restoration (Figure 6I). Together, these results suggest the presence of at least two parallel and mechanistically distinct senescence programs in cardiomyocyte.

**FIGURE 6:**
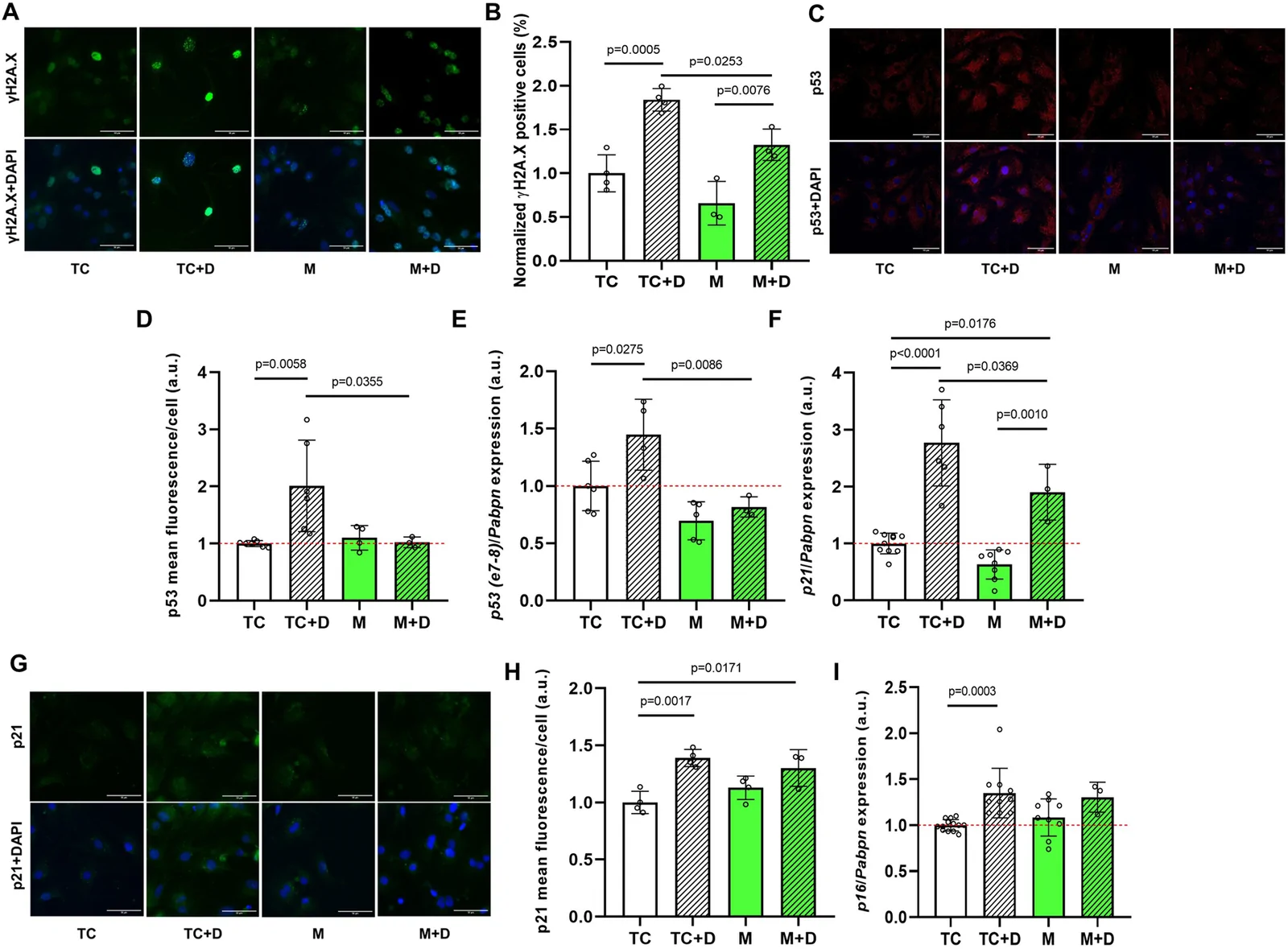
Effect of prophylactic miR-15b restoration on the γH2AX/p53/p21/p16 axis DOX-induced in NRVMs. **(A,B)** Representative immunofluorescence images and quantification of nuclear γH2AX fluorescence intensity (n = 3–4). **(C,D)** Representative immunofluorescence images and quantification of nuclear p53 fluorescence intensity (n = 3–7). **(E)** *p53* mRNA (n = 3–6) and **(F)** *p21* mRNA (n = 3–9) were measured by RT-qPCR. **(G,H)** Representative immunofluorescence images and quantification of nuclear p21 fluorescence intensity (n = 3–4). **(I)** *p16* mRNA (n = 3–9). mRNA levels were normalized to *Pabpn* mRNA. Data are presented as mean ± standard deviation (SD). Comparisons among 4 groups were evaluated by one-way ANOVA followed by Tukey’s *post hoc* test. p < 0.05 was considered statistically significant difference. TC: transfection control; M: miR-15b mimetic (25 nM); TC + D: DOX (0.1 µM); M + D: combined mimetic + DOX.

In addition, while DOX increased the proportion of SA-β-gal-positive cells, this effect was attenuated by pre-transfection with the miR-15b mimic (Figures 7A,B), confirming the rescue of the canonical senescent phenotype. Beyond canonical senescence, miR-15b restoration also attenuated key SASP components: DOX-induced *Il-6* (Figure 7C) and *Il-1β* (Figure 7D) mRNA elevations were completely abrogated in M + D cells, suggesting that the miR-15b/SIRT4 axis contributes to the paracrine inflammatory output of senescent cardiomyocytes. Conversely, other SASP components, such as *Mcp-1* (Figure 7E) and *Mmp13* (Figure 7F), were not reversed by miR-15b pre-transfection. Notably, *Tgf-β* exhibited only a trend toward elevation following DOX treatment (p = 0.0930, Figure 3E); nevertheless, its levels were significantly reduced upon pre-transfection with miR-15b (Figure 7G).

**FIGURE 7:**
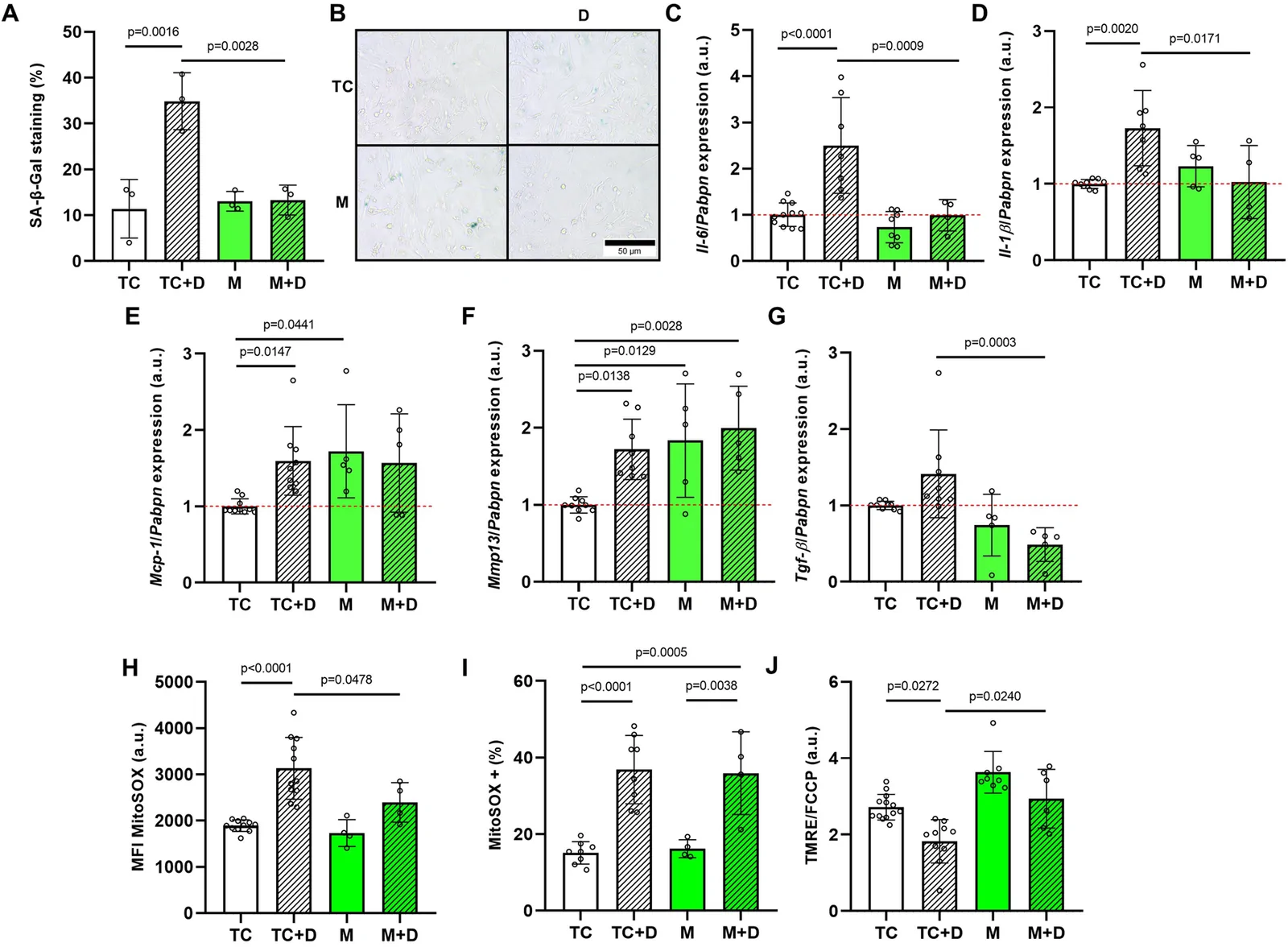
Effect of prophylactic miR-15b on other markers of senescence in DOX-treated NRVMs. **(A,B)** Quantification of the Lysosomal function by peri-nuclear β-Gal staining (n = 3–5). **(C)** *Il-6* mRNA (n = 4–13), **(D)** *Il-1β* mRNA (n = 4–9), **(E)** *Mcp-1* mRNA (n = 5–10), **(F)** *Mmp13* mRNA (n = 5–9), and **(G)** *Tgf-β* mRNA (n = 5–9) were measured by RT-qPCR. **(H,I)** Quantification of the MitoSOX by flow cytometry (mitochondrial superoxide, n = 4–11). **(J)** TMRE fluorescence normalized to MitoTracker (ΔΨm, n = 6–13) by flow cytometry. mRNA levels were normalized to *Pabpn* mRNA. Data are presented as mean ± standard deviation (SD). Comparisons among 4 groups were evaluated by one-way ANOVA followed by Tukey’s *post hoc* test. p < 0.05 was considered statistically significant difference. TC: transfection control; M: miR-15b mimetic (25 nM); TC + D: DOX (0.1 µM); M + D: combined mimetic + DOX.

Consistent with findings pointing to a partial reversal of the SIPS phenotype, prophylactic miR-15b overexpression significantly reduced mitochondrial superoxide levels (MitoSOX; Figure 7H), although not the percentage of cells with elevated superoxide relative to the total population (Figure 7I). Furthermore, DOX-induced ΔΨm dissipation was significantly attenuated in M + D cardiomyocytes (Figure 7J), indicating that the miR-15b/SIRT4 axis also contributes to DOX-induced mitochondrial dysfunction.

### 3.4 Chronic *in vivo* DOX administration stratifies murine survival and induces atrophic myocardial remodeling

To validate the physiological relevance of the miR-15b/SIRT4 axis in a chronic *in vivo* context, we implemented a preclinical model of chronic anthracycline cardiotoxicity in female C57BL/6 mice. Animals received weekly intraperitoneal injections of 6 mg/kg DOX for 5 consecutive weeks (cumulative dose: 30 mg/kg), followed by a 5-week monitoring period (Figure 8A). Body weight in DOX-treated animals stagnated relative to initial values, without the progressive increase observed in controls, and this divergence was maintained throughout the monitoring period (Figure 8B). This was accompanied by a significant reduction in the heart weight-to-tibia length (HW/TL) ratio in DOX-treated animals compared to controls (Figure 8C), consistent with DOX-induced cardiac atrophy.

**FIGURE 8:**
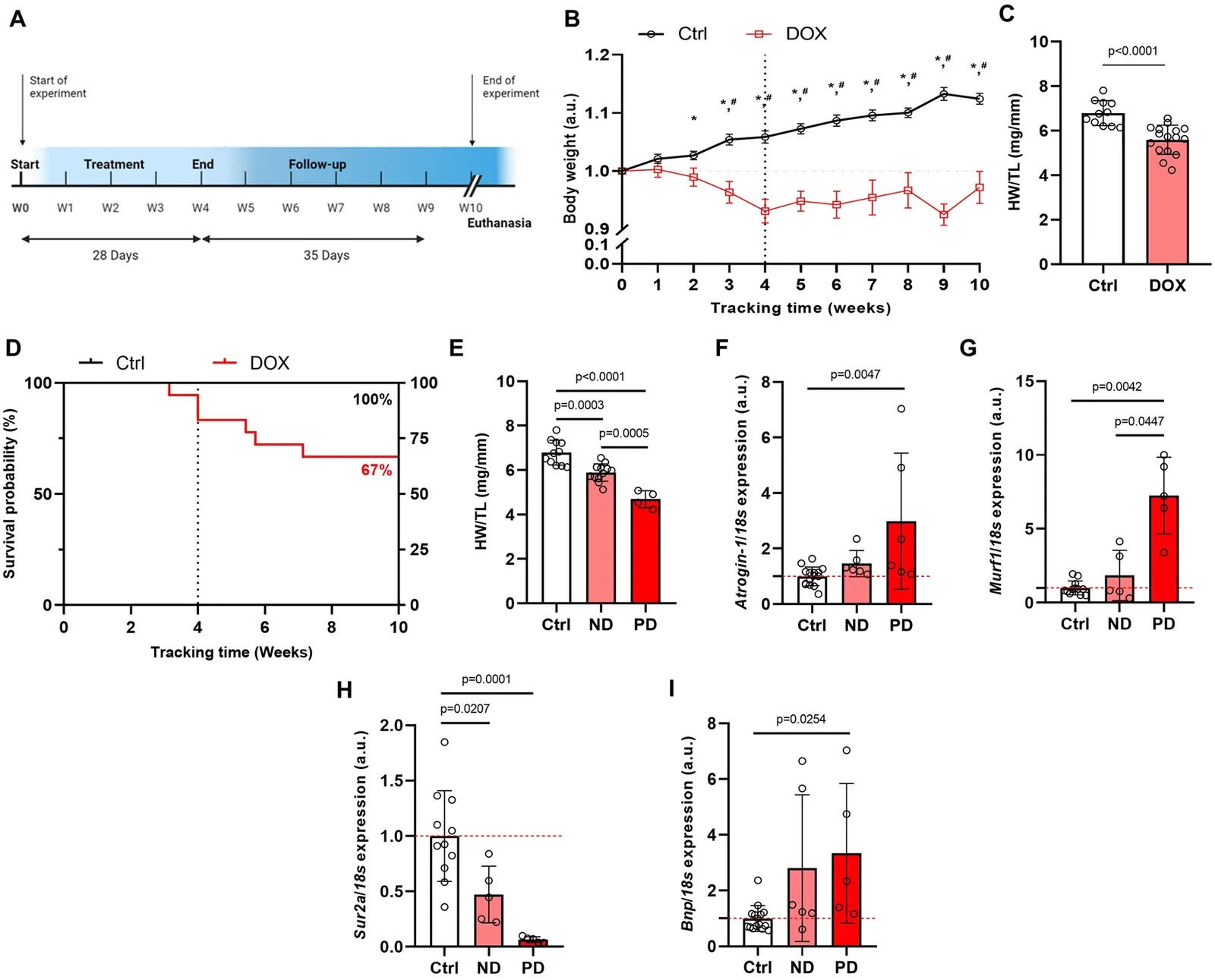
Chronic *in vivo* DOX administration protocol, survival stratification, and myocardial remodeling in female C57BL/6 mice. **(A)** Schematic of the experimental protocol (5 weeks of treatment followed by 5 weeks of monitoring). **(B)** Longitudinal normalized body weight curves, Ctrl vs. DOX (n = 11). **(C)** Heart weight-to-tibia length (HW/TL) ratio, Ctrl vs. DOX (n = 11–16). **(D)** Kaplan–Meier survival curve; log-rank (Mantel–Cox) test; DOX-treated cohort stratified into PD and ND subgroups. **(E)** HW/TL ratio across Ctrl, ND, and PD groups (n = 4–12). **(F)** *Atrogin-1* mRNA (n = 5–12). **(G)** *Murf1* mRNA (n = 5–12). **(H)** *Sur2a* mRNA (n = 5–12) and, **(I)** *Bnp* mRNA (n = 5–12) by RT-qPCR. mRNA levels were normalized to 18S rRNA. Data are presented as mean ± standard deviation (SD). Comparisons among three or more groups were evaluated by one-way ANOVA (or two-way ANOVA where indicated) followed by Tukey’s *post hoc* test. Comparisons between two groups were evaluated by Student’s t-test or the Mann–Whitney test depending on data distribution. p < 0.05 was considered statistically significant difference. Ctrl: control (saline); DOX: doxorubicin; ND: DOX-treated animals that completed the full protocol; PD: DOX-treated animals with premature death.

Kaplan–Meier survival analysis (Figure 8D) revealed that chronic DOX administration profoundly stratified the experimental cohort into two phenotypically distinct subgroups. A minority subgroup (33% of the DOX-treated cohort, designated PD, for premature death) experienced clear signs of distress, such as shortness of breath, piloerection, and immobility, followed by early lethality during or shortly after the treatment phase, while the remaining animals (67%, designated ND, for normal death) successfully completed the full 10-week protocol. The PD subgroup exhibited a significantly lower HW/TL ratio than the ND group (Figure 8E), providing an anatomic correlate of differential cardiotoxicity severity. Consistently, the E3 ubiquitin ligase transcripts *Atrogin-1* (MAFbx/FBXO32) and *MuRF1* (TRIM63) were significantly upregulated in the myocardium of DOX-treated animals (Figures 8F,G), supporting the associated molecular remodeling. *Sur2a* (Abcc9), a gene whose downregulation is associated with impaired metabolic stress adaptation, was significantly reduced, while *Bnp* (Nppb), a marker of ventricular wall stress, was significantly elevated in DOX-treated animals (Figures 8H,I). These findings establish that the chronic DOX protocol in female C57BL/6 mice recapitulates key features of anthracycline cardiomyopathy and generates phenotypic heterogeneity mirroring the clinical variability observed in patients.

### 3.5 Myocardial miR-15b suppression and its target expression profile are associated with the worst *survival* phenotype *in vivo*

To determine whether the miR-15b regulatory axis identified *in vitro* was operative *in vivo*, we systematically evaluated myocardial miR-15b and target mRNA expression profiles across the three experimental groups (Ctrl, ND, PD). RT-qPCR quantification of mature miR-15b revealed a divergent, survival-subgroup-specific pattern: whereas the ND group showed a non-significant trend toward elevated miR-15b relative to controls, the PD group exhibited a pronounced and statistically significant reduction in myocardial miR-15b levels compared to the ND group (p = 0.0091; Figure 9A). This finding suggests that the capacity to maintain or restore myocardial miR-15b may be a determinant of adaptive reserve against anthracycline cardiotoxicity. The pre-miR-15b transcript showed a parallel pattern, with the ND group exhibiting significantly higher precursor levels than controls and the PD group showing significantly lower levels than ND (Figure 9B), pointing to a transcriptionally regulated divergence in miR-15b biogenesis between the two DOX-response phenotypes. Consistently, *Smc4*—the host gene for the miR-15b-encoding intron—showed a significant decrease in the PD group relative to ND (Figure 9C).

**FIGURE 9:**
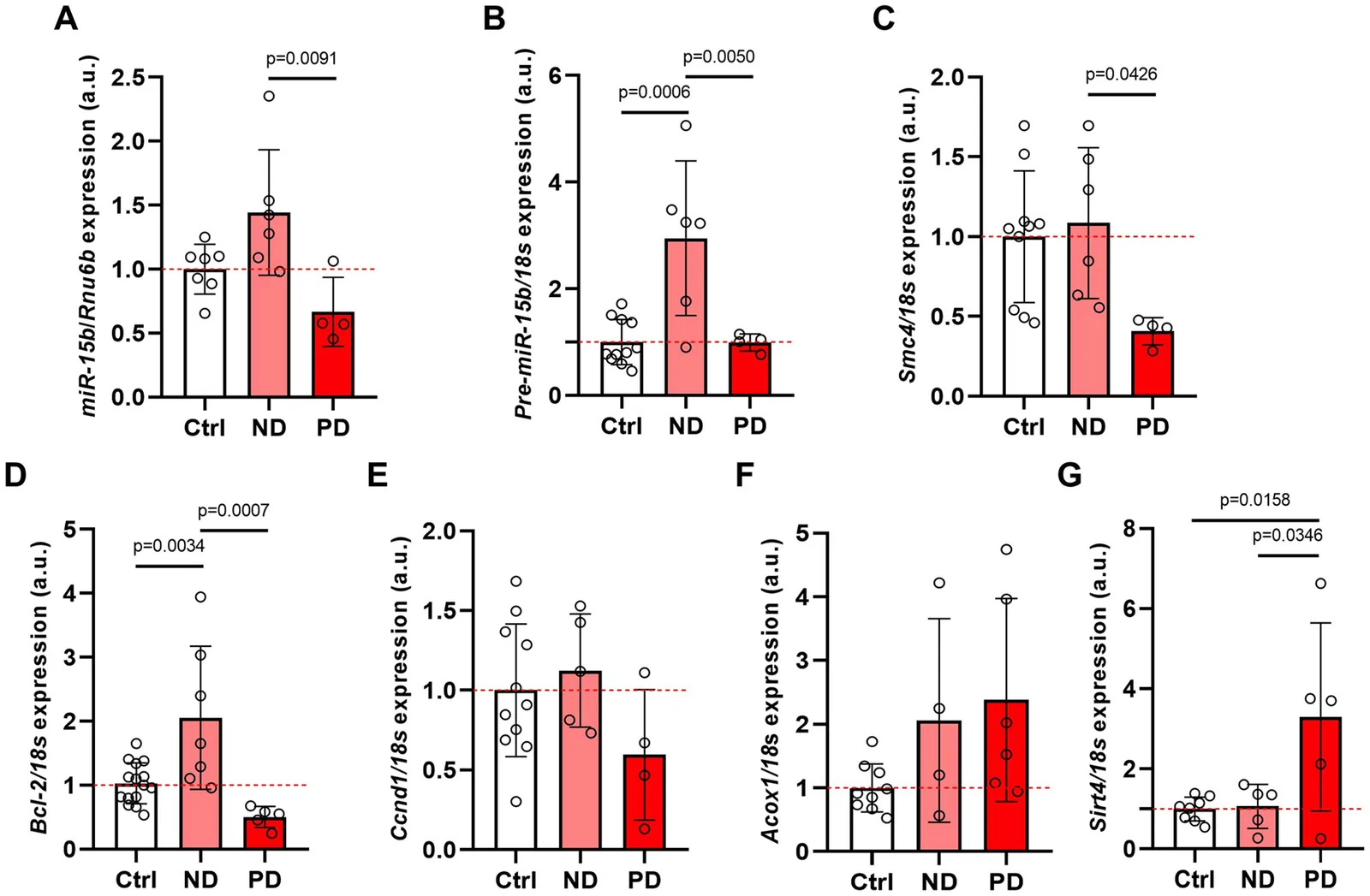
Myocardial miR-15b and target mRNA expression profiles. **(A)** Mature miR-15b normalized to *RnU6b* (n = 4–7), **(B)** Pre-miR-15b normalized to *18S* rRNA (n = 4–11), **(C)** *Smc4* mRNA (n = 4–10), **(D)** *Bcl-2* mRNA (n = 5–15), **(E)** *Ccnd1* mRNA (n = 4–12), **(F)** *Acox1* mRNA (n = 4–9), and **(G)** *Sirt-4* mRNA (n = 5–8) were measured by RT-qPCR. mRNA levels were normalized to 18S rRNA. Data are presented as mean ± standard deviation (SD). Comparisons among 3 groups were evaluated by one-way ANOVA followed by Tukey’s *post hoc* test. p < 0.05 was considered statistically significant difference. ns: statistically non-significant difference. Ctrl: control (saline); DOX: doxorubicin; ND: DOX-treated animals that completed the full protocol; PD: DOX-treated animals with premature death.

Among the miR-15b targets evaluated, *Bcl-2* mRNA showed a significant increase in the ND group relative to controls and a significant decrease in the PD group relative to ND (Figure 9D), whereas *Ccnd1* and *Acox1* mRNA levels showed no statistically significant differences across groups (Figures 9E,F). Conversely, myocardial *Sirt4* mRNA expression was significantly derepressed in DOX-treated mice, with the most pronounced elevation observed in the PD subgroup (Figure 9G). Consistent with these target alterations, myocardial miR-15b levels showed the deepest suppression in the PD group. This inverse correlation between miR-15b expression and cardiotoxicity severity provides *in vivo* evidence that miR-15b dysregulation directly reflects and potentially determines the magnitude of myocardial injury, rather than acting as a mere epiphenomenon of DOX exposure.

### 3.6 Accumulation of SIPS markers with a qualitatively distinct SASP signature is associated with the most severe cardiotoxic response *in vivo*

To comprehensively characterize the *in vivo* senescence landscape associated with differential survival, the full spectrum of SIPS markers, encompassing DNA damage signaling, cell cycle arrest pathways, nuclear envelope integrity, and the SASP, was evaluated at protein and mRNA levels in cardiac tissue from Ctrl, ND, and PD groups. Immunoblot analysis showed that myocardial γH2AX protein content was significantly elevated only in PD animals (Figure 10A). At the transcriptional level, *p53* mRNA displayed a divergent pattern, with a significant increase in the ND group but a decrease in the PD group relative to both ND and Ctrl (Figure 10B). At the protein level, however, p53 was elevated in both DOX-treated groups relative to controls (Figure 10C). *p21* mRNA (Figure 10D) and protein (Figure 10E) were significantly elevated in both DOX groups relative to controls. Notably, *p16* mRNA showed a marked and statistically significant increase selectively in the PD group compared to both Ctrl and ND (Figure 10F), consistent with greater engagement of the p16/Rb pathway in the worst-prognosis animals. Lamin B protein was significantly reduced in both DOX groups, with a more pronounced loss in the PD group (Figure 11A), confirming progressive deterioration of nuclear envelope integrity as a shared but severity-dependent consequence of anthracycline exposure.

**FIGURE 10:**
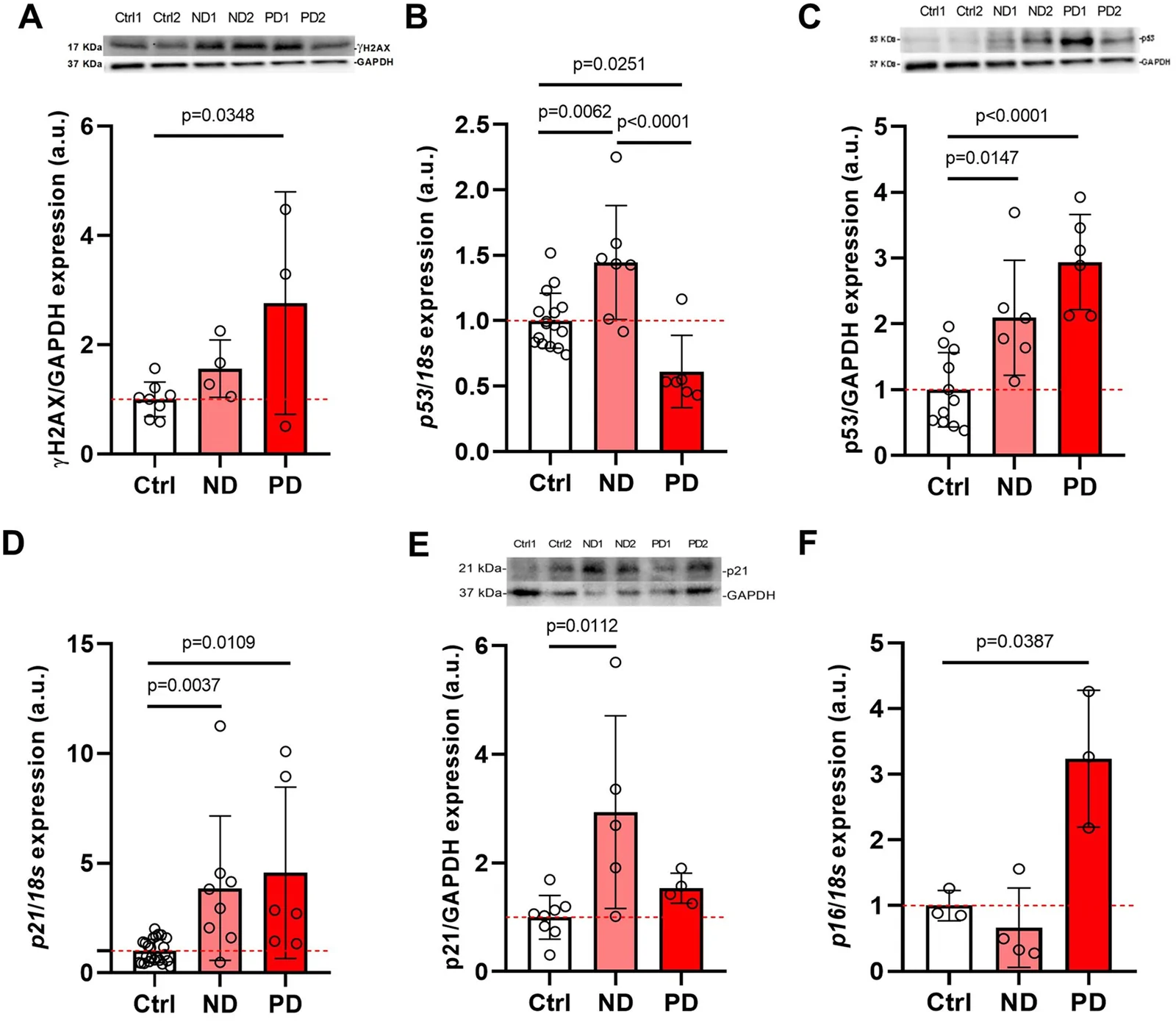
Activation of the γH2AX/p53/p21/p16 axis in DOX-treated mice *in vivo*. **(A)** γH2AX protein normalized to GAPDH, by immunoblot (n = 3–8). **(B)** *p53* mRNA by RT-qPCR (n = 6–16). **(C)** p53 protein normalized to GAPDH, by immunoblot (n = 6–11). **(D)** *p21* mRNA (n = 6–24) by RT-qPCR. **(E)** p21 protein normalized to GAPDH, by immunoblot (n = 4–8). **(F)** *p16* mRNA (n = 3–4) by RT-qPCR. mRNA levels were normalized to 18S rRNA. Data are presented as mean ± standard deviation (SD). Comparisons among 3 groups were evaluated by one-way ANOVA followed by Tukey’s *post hoc* test. p < 0.05 was considered statistically significant difference. ns: statistically non-significant difference. Ctrl: control (saline); DOX: doxorubicin; ND: DOX-treated animals that completed the full protocol; PD: DOX-treated animals with premature death.

**FIGURE 11:**
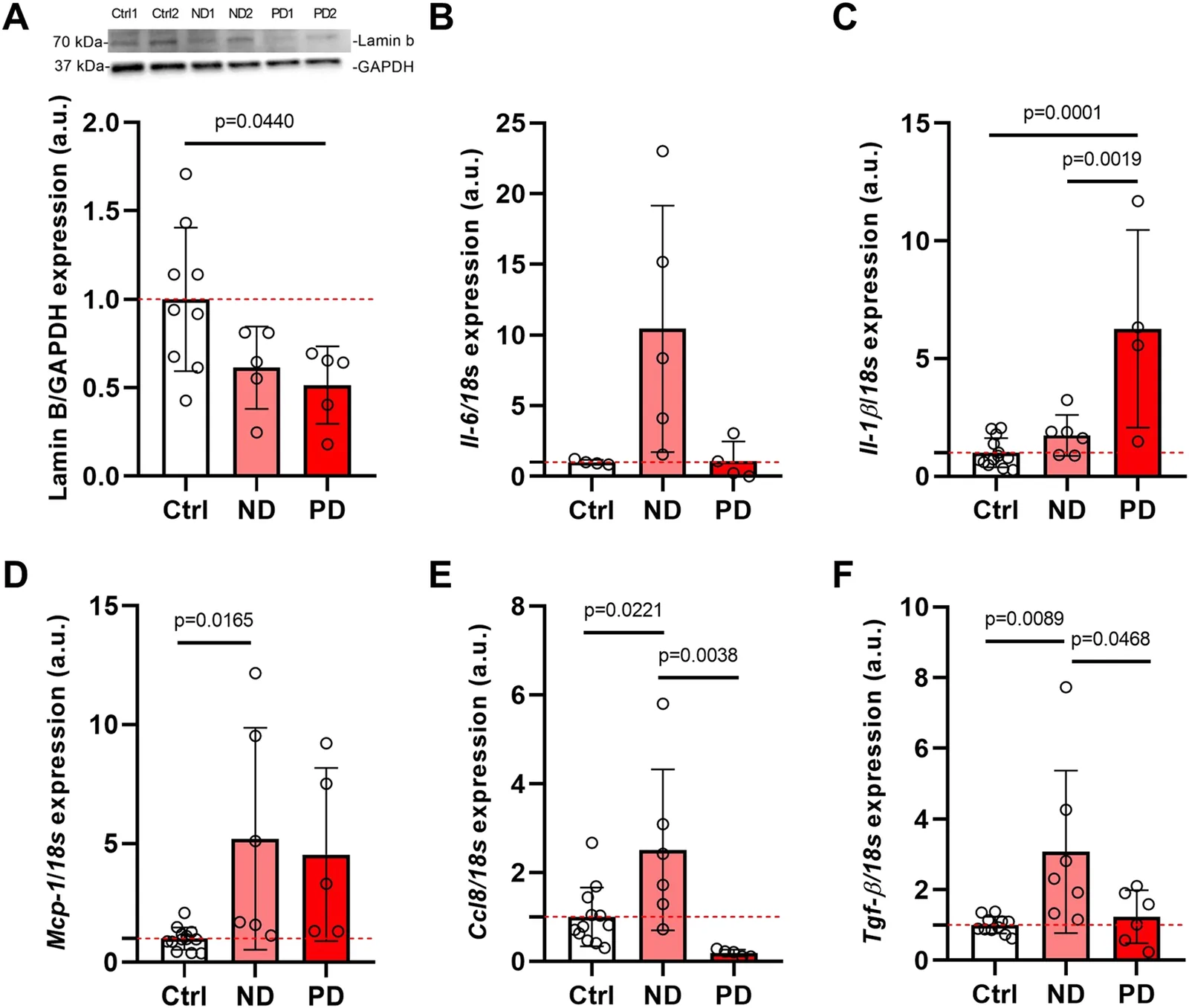
Myocardial SIPS markers. **(A)** Lamin B protein normalized to GAPDH, by immunoblot (n = 5–9). **(B)** *Il-6* mRNA (n = 4–5), **(C)** *Il-1β* mRNA (n = 4–13), **(D)** *Mcp1* (*Ccl2*) mRNA (n = 5–13), **(E)** *Ccl8* mRNA (n = 5–12), and **(F)** *Tgf-β* mRNA (n = 6–11) were measure by RT-qPCR. mRNA levels were normalized to 18S rRNA. Data are presented as mean ± standard deviation (SD). Comparisons among 3 groups were evaluated by one-way ANOVA followed by Tukey’s *post hoc* test. p < 0.05 was considered statistically significant difference. ns: statistically non-significant difference. Ctrl: control (saline); DOX: doxorubicin; ND: DOX-treated animals that completed the full protocol; PD: DOX-treated animals with premature death.

SASP characterization revealed a qualitative bifurcation that discriminated between the two DOX-treated subgroups: *Il-6* mRNA levels were unchanged across groups (Figure 11B), whereas *Il-1β* mRNA was robustly and significantly elevated in the PD group but not in ND (Figure 11C). *Mcp-1* (CCL2) mRNA was elevated in the ND group relative to controls (Figure 11D), establishing it as a shared SASP component irrespective of disease severity. Conversely, *Ccl8* mRNA displayed the opposite pattern, with significant elevation in ND but marked suppression in PD relative to both Ctrl and ND (Figure 11E); *Tgf-β* exhibited the same profile (Figure 11F). Together, these results suggest that SIPS severity and SASP composition act in concert with miR-15b levels to define the *in vivo* trajectory of chronic DOX cardiotoxicity.

## 4 Discussion

This study provides mechanistic evidence that miR-15b acts as a post-transcriptional regulator of stress-induced premature senescence (SIPS) in cardiomyocytes exposed to doxorubicin (DOX), and that cardiac miR-15b levels are inversely associated with the severity of chronic cardiotoxicity *in vivo*. Collectively, our data support a model in which DOX, at clinically relevant concentrations, suppresses miR-15b, derepresses SIRT4, and activates the p53/p21 pathway, suggesting a direct contribution to a senescent program that perpetuates pro-inflammatory cardiac remodeling in female mice.

### 4.1 Reduction of miR-15b as a permissive mechanism for DOX-induced SIPS

Our findings suggest that miR-15b downregulation is a permissive event in DOX-induced SIPS. In neonatal rat ventricular myocytes (NRVMs), 0.1 µM DOX for 24 h reduced miR-15b by approximately 50%, consistent with the low-dose effects reported by Holmgren et al. (2016) in human iPSC-derived cardiomyocytes, but in contrast to the increase observed at much higher DOX concentrations by Wan et al. (2019) and Wan et al. (2021). This apparent discrepancy likely reflects dose- and context-dependent responses, since the concentrations used by Wan et al. (2019) and Wan et al. (2021) exceed clinically achievable plasma peaks, whereas 0.1 µM is sufficient to induce senescence without overt cell death (Spallarossa et al., 2009; Harahap et al., 2020; Eksborg et al., 1985). By targeting this clinically relevant range, our findings align with the concept that chronic anthracycline cardiotoxicity is driven primarily by progressive cardiomyocyte dysfunction rather than massive cell loss (Zhang et al., 2012; Zeiss et al., 2019). Moreover, recent evidence has shown that senolytics can prevent or reverse the cardiotoxic effects of DOX at the cellular level (Xia et al., 2024; Scalise et al., 2025) and in murine models (Xia et al., 2024; Lérida-Viso et al., 2022), further positioning premature senescence as a key determinant of this phenotype at therapeutic doses.

In line with this model, and although we were unable to measure SIRT4 protein levels, our data indicate that miR-15b reduction is associated with *Sirt4* mRNA derepression, an effect partially prevented by prophylactic transfection with the miR-15b mimic. This regulatory relationship has previously been described in senescent human dermal fibroblasts, where miR-15b targets the 3′UTR of *Sirt4* (Lang et al., 2016). SIRT4 has been linked to mitochondrial dysfunction, ROS production, and SASP establishment, supporting the idea that the miR-15b/SIRT4 axis is a conserved regulator of mitochondrial homeostasis and senescence across cell types. Thus, the partial rescue observed with miR-15b overexpression suggests that loss of miR-15b is an important, though not exclusive; step in DOX-induced mitochondrial dysregulation and SIPS. In our study, a prophylactic transfection strategy (miR-15b mimetic administration prior to DOX challenge) was chosen to interrogate the mechanistic necessity of miR-15b loss in permissive SIPS execution. From a translational perspective, future studies employing post-treatment administration schedules via cardiotropic vectors such as AAV9 will be essential to evaluate whether restoring miR-15b can reverse or arrest ongoing anthracycline-induced cardiotoxicity in established disease.

### 4.2 miR-15b modulates p53/p21 but not p16

Preventive restoration of miR-15b selectively attenuated p53/p21 activation but did not affect the p16/Rb pathway, suggesting that DOX activates at least two parallel senescence programs in cardiomyocytes: a DNA damage–dependent ATM/CHK2/p53/p21 axis, at least partly controlled by the miR-15b/SIRT4 pathway, and a p16-dependent program, potentially mediated by GATA4/NF-κB signaling or DOX-induced epigenetic dysregulation (Pommier et al., 2016; Kang et al., 2015; Ohtani and Hara, 2009). miR-15b may regulate p53 indirectly, as reported in other contexts, possibly through MALAT1, HOTAIR, or related regulators, although the mechanism in cardiac tissue remains unknown (Sun et al., 2018; Rahman et al., 2014; Yang et al., 2022; Cai et al., 2019; Li and Wang, 2022; Liu et al., 2025). The inabilities to suppress p16 suggest that miR-15b alone is insufficient to fully reverse DOX-induced SIPS. A clear limitation of our study is that the proposed involvement of GATA4 and NF-κB in this p16-dependent program remains speculative and was not experimentally validated; warranting targeted mechanistic studies in the future.

miR-15b is reduced in senescent fibroblasts from different tissues, supporting its role in conserved senescence pathways (Holly et al., 2015). In addition, extracellular vesicles enriched in miR-15b can partially reverse senescence in human fibroblasts, and miR-15b functions as a mito-miR that directly targets mitochondrial genes such as mitofusin-2 (Mfn2), linking it to mitochondrial stress and dysfunction (Dasgupta et al., 2015; Nishi et al., 2010; Yu et al., 2023; Zhang et al., 2024). Overexpression of miR-15b-5p also alters the cardiac proteome and impairs mitochondrial stability and fatty acid metabolism, which could be detrimental at pro-senescent levels (Pantaleão et al., 2024). Furthermore, given that SIRT4 and microRNA pathways can intersect with metabolic and autophagic regulation, and considering the complex, double-edged role of autophagy in DOX-induced cardiotoxicity (Christidi and Brunham, 2021), whether the miR-15b/SIRT4 axis modulates autophagic flux to influence senescence represents an important avenue for future research.

### 4.3 Sexual dimorphism in susceptibility to DOX cardiotoxicity

The female C57BL/6 mouse model required a cumulative DOX dose of 30 mg/kg to induce cardiotoxicity, higher than that typically used in male models (Podyacheva et al., 2021). This greater resistance in females is consistent with preclinical evidence of sex differences in cardiac cytochrome P450 enzymes that metabolize DOX: males preferentially express Cyp1b1, whereas females express Cyp2c29 and Cyp2e1, isoforms that more efficiently metabolize the anthracycline (Grant et al., 2017; Grant et al., 2019; Grant et al., 2020). Sexual dimorphism has also been reported in cardiac mitochondrial oxidative gene expression (Gonzalez et al., 2015) and in the remodeling of myocardial lipid metabolism in response to DOX (Moulin et al., 2015). The use of a female model is clinically relevant because breast cancer, the most common indication for DOX, occurs predominantly in women, and female preclinical studies have been historically limited. Thus, our model helps address this gap, although the results should be interpreted in light of sex-related cardioprotective mechanisms that may influence the magnitude and phenotype of SIPS.

### 4.4 Heterogeneity of the *in vivo* response: ND and PD subgroups

One of the most relevant findings of our *in vivo* model is the segregation of DOX-treated animals into two subgroups with distinct pathological severity, recapitulating the clinical variability observed in humans, where the incidence of DOX-induced cardiomyopathy ranges from 4% to 46% depending on cumulative dose (Lefrak et al., 1973). Morphologically, the PD group showed more severe cardiac atrophy, with a greater reduction in the cardiac weight/tibial length ratio and higher Atrogin-1 and MuRF1 levels than the ND group. This suggests that the adaptive capacity of the cardiomyocyte (Willis et al., 2019), at least in part reflected by relative preservation of miR-15b in the ND group, modulates the extent of structural remodeling. Consistently, the PD group exhibited a stronger accumulation of SIPS markers, including increased p53/p21 and p16 activation, more γH2AX foci, greater Lamin B loss, and more pronounced mitochondrial dysfunction, supporting a model in which deeper miR-15b suppression perpetuates SIRT4-dependent mitochondrial dysfunction, amplifies genotoxic damage, and drives a more severe senescent phenotype.

miR-15b may also have a context-dependent role in cardiac remodeling. It has been described as part of the cardiac stress-response signature and is overexpressed during hypertrophy and heart failure (Van Rooij et al., 2006; Hu et al., 2025). In contrast, our chronic DOX model demonstrates miR-15b suppression associated with atrophy rather than hypertrophy, suggesting that miR-15b exerts context-dependent effects in the heart: its upregulation may favor pathological hypertrophy and apoptosis (Hullinger et al., 2012), whereas its chronic downregulation may promote senescence and atrophy.

### 4.5 miR-15b as a severity marker

The inverse relationship between myocardial miR-15b levels and disease severity suggests it may serve as a tissue-level correlate of chronic DOX cardiotoxicity, aligned with reported miRNA dynamics in cardiac injury (Holmgren et al., 2016; Ruggeri et al., 2018a; Ruggeri et al., 2018b). Contextualizing miR-15b across cardiac pathologies highlights its context-dependent nature: while acute overexpression drives apoptosis and exacerbates ischemia-reperfusion injury through Bcl-2 and PERK pathways (Hullinger et al., 2012; Liu et al., 2012), its chronic downregulation—as seen in diabetic cardiomyopathy (Rawal et al., 2017) and our DOX model—correlates with fibrotic and atrophic remodeling. Together, these patterns support a model wherein acute induction vs. chronic suppression of miR-15b yields distinct cell-fate outcomes. Preserving basal miR-15b levels may thus be relevant to maintaining cardiac homeostasis during stress (Smith-Vikos et al., 2016).

The inability of the PD subgroup to maintain miR-15b during chronic DOX exposure may reflect reduced mitochondrial resilience, thereby perpetuating dysfunction through the miR-15b/SIRT4 axis and amplifying the SASP. Future studies will be necessary to determine whether sustained miR-15b reduction, rather than early suppression, is the key determinant of severe responses. In addition, *in vivo* gain- and loss-of-function studies using cardiotropic vectors will be required to establish causality between miR-15b loss and chronic cardiotoxicity.

### 4.6 The dynamics of p53, p21, and p16 as the key to differences between subgroups

The apparent discrepancy between p53 mRNA, which decreased in PD relative to ND, and p53 protein, which increased in both DOX groups, likely reflects the post-translational dynamics and temporal regulation of p53 during chronic genotoxic stress. Nevertheless, time-course kinetics will be required in future studies to confirm this possibility. Regardless, p53 is known to undergo transient pulses of activation followed by degradation (El-Deiry et al., 1993; El-Deiry et al., 1994; L’Ecuyer et al., 2006), whereas p21 tends to exhibit a more stable and sustained expression once senescence is established (Hsu et al., 2019). Thus, p53 transcriptional activity may rise over time without a parallel increase in total protein, while remaining sufficient to sustain p21 expression (Vaziri et al., 1997; Lieschke et al., 2024). In the PD subgroup, deeper miR-15b suppression and greater mitochondrial dysfunction may sustain chronic p53 activation, whereas the ND group may exhibit more transient signaling or a compensatory phenotype that supports survival. The selective increase in p16 in the PD group adds another layer of complexity, since the p16/Rb pathway acts independently of p53 (Engeland, 2022) and may convert the more tolerable senescent state of the ND group into the maladaptive senescence observed in PD, helping to explain the SASP bifurcation.

### 4.7 SASP bifurcation as a determinant of clinical outcome

The ND and PD subgroups differed not only in SASP magnitude but also in cytokine composition, yielding diametrically opposed signatures. The MCP-1-high/IL-1βhigh/CCL8-low profile of the PD group, dominated by IL-1β, is consistent with acute inflammatory activation mediated by the NLRP3 inflammasome, which can amplify senescence in an autocrine and paracrine manner and has been linked to maladaptive remodeling, fibrosis, and diastolic dysfunction (Mo et al., 2025). Similar senescence-associated inflammatory programs have been described in sepsis-induced cardiac injury, where Nppb-regulated networks upregulate IL-1β and accelerate cardiomyocyte decline (Yang et al., 2024). In this setting, sustained miR-15b loss may derepress SIRT4, increase mitochondrial ROS, and activate the cGAS-STING-NF-κB axis, ultimately promoting robust NLRP3 inflammasome activation and excessive IL-1β release.

By contrast, the MCP-1-high/IL-1βlow/CCL8-high signature of the ND group may represent a more contained, potentially adaptive inflammatory response. It should be noted that the characterization of *Il1b* and *Ccl8* dynamics in this model reflects transcriptional induction profiles; future studies utilizing protein-level assays (e.g., ELISA) will be valuable to confirm their final secretion kinetics.

Such qualitative SASP heterogeneity has been reported in oncogene activation, SIPS, pharmacological stress, and tissue-specific contexts (Basisty et al., 2020; Suryadevara et al., 2024), but its specific configuration in DOX-induced cardiotoxicity has not been previously described. CCL8, which was selectively elevated in ND, has broader receptor promiscuity than MCP-1 and can bind CCR1, CCR2, CCR3, and CCR5, receptors expressed on NK cells, Tregs, and M2-polarized macrophages (Hughes and Nibbs, 2018). This profile suggests recruitment of immunomodulatory and tissue-reparative populations rather than purely pro-inflammatory infiltration, although CCL8 may still contribute to remodeling (Amador-Martínez et al., 2021; Yan et al., 2026; Gorgoulis et al., 2019). MCP-1, in contrast, is a canonical monocyte-chemoattracting chemokine that promotes recruitment of monocytes and macrophages to injured myocardium and is one of the earliest inflammatory signals after DOX exposure (Zhang et al., 2022; Gao et al., 2026). Its elevation in both groups indicates that MCP-1 is a universal component of anthracycline-induced SASP, whereas the CCL8 rise in ND may reflect a compensated inflammatory phenotype. Notably, recent evidence also indicates that CCL2/MCP-1 can support tissue regeneration in some settings, reinforcing the idea that the ND SASP is not necessarily non-remodeling, but rather less maladaptive.

### 4.8 miR-15b in the oncological context: duality and tissue context

The role of miR-15b in oncology is clearly context-dependent: it acts as a tumor suppressor in hematological malignancies (Lovat et al., 2018), is associated with chemoresistance in gastric cancer (Xia et al., 2008), and behaves as an oncogene in bladder, breast, and prostate cancer (Chen et al., 2018; Wu et al., 2020). Although our *in vitro* rescue experiments provide mechanistic causality in isolated cardiomyocytes, confirming whether targeted myocardial miR-15b overexpression via AAV9 vectors rescues chronic mortality *in vivo* remains an essential objective for subsequent studies. Moreover, in cardiomyocytes, our finding that miR-15b restoration suppresses senescence is consistent with a protective role against a harmful phenotype; however, in oncological patients, the same signaling could undesirably interfere with senescence in residual tumor cells. This paradox highlights the need for cardiac-specific delivery strategies before considering clinical translation.

This issue is further complicated by recent evidence showing that simultaneous inhibition of miR-1a and miR-15b with locked nucleic acid molecules (LNA-1a/15b) promotes adult cardiomyocyte proliferation, improves contractility in human cardiac organoids, and reduces post-infarction damage in mice (Yuan et al., 2025). Because miR-15b is a central suppressor of postnatal cardiomyocyte mitotic arrest (Porrello et al., 2011), its inhibition could in principle reactivate a proliferative program in DOX cardiotoxicity. However, this is the opposite of the miR-15b restoration strategy proposed here to limit senescence, underscoring the functional duality of this miRNA: inhibition may favor regeneration after infarction, whereas chronic loss may promote pathological senescence. This tension must be resolved before miR-15b-based therapies can be applied in anthracycline-treated patients.

Although neonatal cardiomyocytes differ from adult ventricular myocytes in their metabolic substrate utilization, electrophysiological maturity, and basal proliferative capacity, in this study we used NRVMs for *in vitro* mechanistic assays because they provide a highly tractable model for dissecting miRNA-mediated post-transcriptional networks and SASP cascades without confounding cell death. Although our key findings were corroborated in adult cardiac tissue *in vivo*, future studies utilizing adult cardiomyocytes will be valuable to further confirm the translational kinetics of the miR-15b/SIRT4 axis in mature human cardiac tissue.

## 5 Conclusion

Taken together, our results demonstrate that DOX suppresses miR-15b in cardiomyocytes, promoting SIRT4-dependent SIPS, and that preventive miR-15b restoration selectively attenuates the p53/p21 axis, DNA damage, and SASP without altering p16/Rb signaling. *In vivo*, chronic DOX exposure reveals a severity-dependent phenotype associated with deeper miR-15b loss and an enhanced pro-inflammatory SASP. While these demonstrated mechanisms establish miR-15b as a critical driver of DOX-induced cardiomyocyte senescence, its potential utility as a therapeutic target or diagnostic biomarker in anthracycline cardiomyopathy represents a prospective clinical implication that warrants future translational validation.

## Data Availability

The raw data supporting the conclusions of this article will be made available by the authors, without undue reservation.
